# Double-Blind Randomized Clinical Trial: Gluten versus Placebo Rechallenge in Patients with Lymphocytic Enteritis and Suspected Celiac Disease

**DOI:** 10.1371/journal.pone.0157879

**Published:** 2016-07-08

**Authors:** Mercè Rosinach, Fernando Fernández-Bañares, Anna Carrasco, Montserrat Ibarra, Rocío Temiño, Antonio Salas, Maria Esteve

**Affiliations:** 1 Department of Gastroenterology, Hospital Universitari Mutua Terrassa, Terrassa, Barcelona, Spain; 2 Centro de Investigación Biomédica en Red de Enfermedades Hepáticas y Digestivas (CIBERehd), Madrid, Spain; 3 Department of Pathology, Hospital Universitari Mutua Terrassa, Terrassa, Barcelona, Spain; University Hospital Llandough, UNITED KINGDOM

## Abstract

**Background:**

The role of gluten as a trigger of symptoms in non-coeliac gluten sensitivity has been questioned.

**Aim:**

To demonstrate that gluten is the trigger of symptoms in a subgroup of patients fulfilling the diagnostic criteria for non-coeliac gluten sensitivity (NCGS), which presented with lymphocytic enteritis, positive celiac genetics and negative celiac serology.

**Methods:**

Double-blind randomized clinical trial of gluten *vs* placebo rechallenge. Inclusion criteria: >18 years of age, HLA-DQ2/8+, negative coeliac serology and gluten-dependent lymphocytic enteritis, and GI symptoms, with clinical and histological remission at inclusion. Eighteen patients were randomised: 11 gluten (20 g/day) and 7 placebo. Clinical symptoms, quality of life (GIQLI), and presence of gamma/delta+ cells and transglutaminase deposits were evaluated.

**Results:**

91% of patients had clinical relapse during gluten challenge versus 28.5% after placebo (p = 0.01). Clinical scores and GIQLI worsened after gluten but not after placebo (p<0.01). The presence of coeliac tissue markers at baseline biopsy on a gluten-free diet allowed classifying 9 out of the 18 (50%) patients as having probable ‘coeliac lite’ disease.

**Conclusion:**

This proof-of-concept study indicates that gluten is the trigger of symptoms in a subgroup of patients fulfilling the diagnostic criteria for NCGS. They were characterized by positive celiac genetics, lymphocytic enteritis, and clinical and histological remission after a gluten-free diet.

**Trial Registration:**

ClinicalTrials.gov NCT02472704

## Introduction

Lymphocytic enteritis (LE) is defined by normal villous architecture and intraepithelial lymphocytes (IEL) >25/100 enterocytes [1]. It is a frequent finding in 2% to 5.4% of duodenal biopsies [2]. LE is secondary to coeliac disease (CoD) only in a minority of patients, since it may be a response to other inflammatory processes in the gut. Other possible aetiologies of LE include infections (*Helicobacter pylori*), drugs (nonsteroidal anti-inflammatory drugs or acetylsalicylic acid) and autoimmune disease [3]. Observational studies have established CoD as accounting for 10% to 43% of cases with LE and positive HLA-DQ2/8 after exhaustive diagnostic work-up [4,5,6]. These ‘minor’ forms of CoD may have similar clinical manifestations to those with villous atrophy [7].

However, these patients with ‘minor’ CoD often have negative coeliac serology, and thus do not fulfil the present criteria to diagnose CoD [8,9]. In fact, using the present diagnostic criteria they should be included in the definition of non-coeliac gluten sensitivity (NCGS) [10]. For a diagnosis of NCGS it is necessary to rule out CoD by means of negative serology—endomysial and tissue transglutaminase IgA antibodies—and a duodenal biopsy with absence of villous atrophy on a gluten-containing diet. As such it is accepted that NCGS patients might have LE [11]. A recent systematic review revealed that 20% of patients with suspected NCGS who are HLA-DQ2/DQ8 positive, whose intestinal biopsy is classified as Marsh stage 1, and who have negative CoD serology, may come within the spectrum now recognised as CoD [12], which some authors have called ‘coeliac lite’ disease [13,14].

In order to ascertain the role of gluten as a trigger of symptoms in patients with presumptive NCGS, several double-blind placebo-controlled dietary interventions have been published [15–19]. The first gluten *vs* placebo rechallenge trial showed that patients who received gluten had significantly more abdominal symptoms than those on placebo (68% *vs* 40%) [15]. The second study that investigated the specific effects of gluten after dietary reduction of fermentable, poorly absorbed, short-chain carbohydrates (FODMAPs) in subjects believed to have NCGS showed no symptomatic worsening after gluten challenge (16 g/d of gluten) as compared to placebo. Thus, there was no evidence of specific or dose-dependent effect of gluten on NCGS patients placed on a diet low in FODMAPs [16]. It is worth mentioning that patients included in these trials were HLA-DQ2/8 negative, and if positive they had a normal duodenal biopsy (Marsh 0) while on a gluten-containing diet. Results of more recent trials suggest that gluten challenge induces symptom recurrence in only a minority of patients who meet clinical criteria for NCGS [17,18]. However, 60% to 70% of patients included in these trials were HLA-DQ2/8 negative, and at least in the Zanini et al study patients classified as Marsh 1 & 2 were included without ruling-out *Helicobacter pylori* infection and other causes of intraepithelial lymphocytosis [17].

The recent ESPGHAN guidelines for CoD diagnosis suggest that in cases with low-grade enteropathy (including LE) both a high γδ IEL count and the presence of IgA anti-tissue transglutaminase (anti-TG2) deposits in the mucosa increase the likelihood of CoD [8]. Thus, these parameters are considered CoD tissue markers. In contrast to CoD, one study suggests that in NCGS there is no increase in T-cell receptor γδ IELs [20]. However, these parameters have only rarely been used to rule out CoD in patients in the literature with NCGS [12].

The aim of the present research was to demonstrate in a ‘proof-of-concept’ study that gluten is the trigger of clinical symptoms in a subgroup of patients fulfilling the present criteria for NCGS. Patients in our study, in contrast to those included in recent trials, were HLA-DQ2/8+, had lymphocytic enteritis, and showed clinical and histological gluten dependency. In addition, the presence of CoD tissue markers while on a GFD was investigated, as a possible biomarker of ‘coeliac lite’ disease.

## Patients and Methods

### Study design

This was a double-blind randomised placebo-controlled clinical trial. Patients were included from February 2012 to December 2013. Last patient ended the follow-up period in May 2014. The study protocol was prepared in accordance with the International Conference on Harmonisation Guidelines for Good Clinical Practice and in full conformity with relevant regulations. The ethical committee of Hospital Universitari Mutua Terrassa approved the study protocol in September 2011. All participants gave written informed consent to participate in the trial. The study protocol was registered at ClinicalTrials.gov with number NCT02472704 in June 2015 (the study protocol, the study database and the Consort 2010 checklist are given S1 and S2 Protocols, S1 File, and S1 CONSORT Checklist). At present, there are no other related trials for this intervention. As mentioned, the trial was designed as a proof-of-concept study to demonstrate that there was a subgroup of patients fulfilling the present criteria of NCGS who, in contrast to others in recent studies [16–18], show a high percentage of gluten-dependency. Since in the Biesiekierski et al study [16], a challenge dose of 16 g of gluten daily had an effect similar to that of placebo, we decided to administer 20 g of gluten daily, taking into account that in Spain a gluten-containing diet has roughly 10 to 40 grams of gluten per day.

### Study population

Men and women aged 18 years or older were eligible for randomization if they had: 1. Presence at initial diagnosis while on a gluten containing diet of: a) LE on distal duodenum biopsy samples defined as IEL >25/100 enterocytes; b) gastro-intestinal clinical symptoms within the clinical spectrum of CoD; c) negative serology for CoD (both serum IgA anti-tissue transglutaminase antibody–anti-tTG2–and anti-endomysium antibodies–EmA) (IgA deficiency was excluded); and d) positive HLA-DQ2.5 and/or HLA-DQ8 haplotypes; 2. Clinical and histological remission after a GFD evaluated after a minimum follow-up of 12 months; and 3. No previous studies of either IEL cytometric pattern or anti-TG2 IgA subepithelial deposits.

At the initial diagnosis, other LE aetiologies, such as non-steroidal anti-inflammatory drug intake, parasitic infection, and *Helicobacter pylori* infection, were appropriately ruled out.

Exclusion criteria were: 1. A previous diagnosis with CoD, i.e., positive coeliac serology and/or villous atrophy; 2. Intake of either non-steroidal anti-inflammatory drugs or olmesartan in the month previous to the inclusion; 3. Presence of parasitic or *Helicobacter pylori* infection; 4. Use of corticosteroids or other immunosupressants for autoimmune associated diseases that could affect the final results; 5. Participation in other randomised controlled trials in the previous four weeks; 6. Inability to adhere to the study visit schedule and other protocol requirements; 7. Other significant intestinal and colonic diseases; 8. Previous gastro-intestinal surgery (except appendectomy and inguinal herniorrhaphy); 9. Severe comorbidities; and 9. Pregnancy or breast-feeding.

### Treatment allocation

A computer-generated list of random numbers was used for allocation of participants. This list was prepared by the Pharmacy Department of Hospital Universitari Mutua Terrassa without any clinical involvement in the trial. Eligible patients were randomly assigned to 1 of 2 treatment groups. The study medication was packed in boxes, and consecutively numbered for each patient according to the randomization schedule. Patients received either 10 g gluten opaque sachets or identical placebo sachets twice daily for 6 months in a double-blind fashion. Participants continued on a GFD throughout the study, and were instructed to mix the gluten or placebo powder with foods (in soup, yogurt, or purees). The 10 g gluten sachets contained 8.1 g gluten, 0.3 g fibre, 1 g fat, and 0.6 g carbohydrate; the placebo sachets contained 10 g maltodextrin (Fagron Ibérica, SA; Terrassa, Spain). There was no FODMAP content in the two types of sachets. The gluten and placebo powders were slightly different in taste but patients ignore how the other formulation was, and it was not possible to recognize gluten containing ones.

### Assessments

Interim visits were made at weeks 2, 4, and 12. Patients unable to continue due to intolerable symptoms after at least 1 week were withdrawn from the study. In all visits patients were asked to complete a clinical symptoms questionnaire containing the questions for the primary outcome detailed below, using a 100-mm visual analogue scale (VAS) [21], with 0 representing no symptoms, which assessed diarrhoea, abdominal pain, abdominal bloating, and flatulence. Thus, overall symptom scores ranged from 0 to 400.

At the beginning and at the end of the study (weeks 0 and 24, or at premature withdrawal), the changes in health-related quality of life (HRQoL) were assessed using the Spanish version of the Gastrointestinal Quality of Life Index (GIQLI) [22]. In addition, blood sampling for EmA and anti-tTG2 assays, and sample biopsies from 2^nd^-3^rd^ portions of the duodenum for histology assessment, and determination of both IEL subpopulations by flow cytometry and anti-tTG2 intestinal deposits, were obtained (see S2 File) [23].

Compliance with the GFD during the trial was assessed by an expert dietician (M.I.) at both baseline and final visit, using analysis of 3-day food records. FODMAP intake was unrestricted. Adherence to the study protocol was monitored by returned unused sachet count at each study visit. During the entire study period, the use of anti-diarrhoeals, spasmolytics, and other drugs causing constipation was not permitted.

### Withdrawal criteria

The reasons for withdrawing a patient early from the study were the following: 1. Intolerable symptoms after at least 1 week of challenge; 2. The need to administer a concomitant medication that was prohibited in the study protocol; 3. Lack of patient cooperation.

### Clinical outcomes evaluation

Our primary end-point was clinical relapse at 6 months, defined as a worsening on any of the symptom scales of more than 40 points over baseline. Secondary end-points were to assess the presence of CoD biomarkers in patients on a GFD (at baseline), and to evaluate changes in overall and individual VAS, GIQLI, serology, histology, and tissue CoD biomarkers.

### Statistical analysis

Sample size was calculated based on the primary end-point clinical relapse rate at 6 months. We hypothesized that gluten-dependency in the selected HLA-DQ2/8+ patients with clinical and histological remission after a GFD would be high. Thus, it was considered that the relapse rate after gluten challenge would be 80%, while after the placebo challenge it would be 15%. Assuming an alpha value of 5% and a 90% statistical power to yield a statistically significant result (Fisher’s exact test), the calculated sample size was 8 patients per group.

Efficacy was analysed for the intention-to-treat (ITT) population. Results are given as mean±Standard error of the mean (SEM) or percentages (and their 95% confidence interval, CI). Fisher’s exact test, paired or unpaired T-test, and Mann-Whitney test were used for the statistical analysis, and type 1 error rate was 2-sided with alpha = 0.05. All statistical analysis was conducted using the SPSS for Windows statistical package (SPSS Inc, Chicago, IL, USA).

## Results

### Patient population

**Fig 1:**
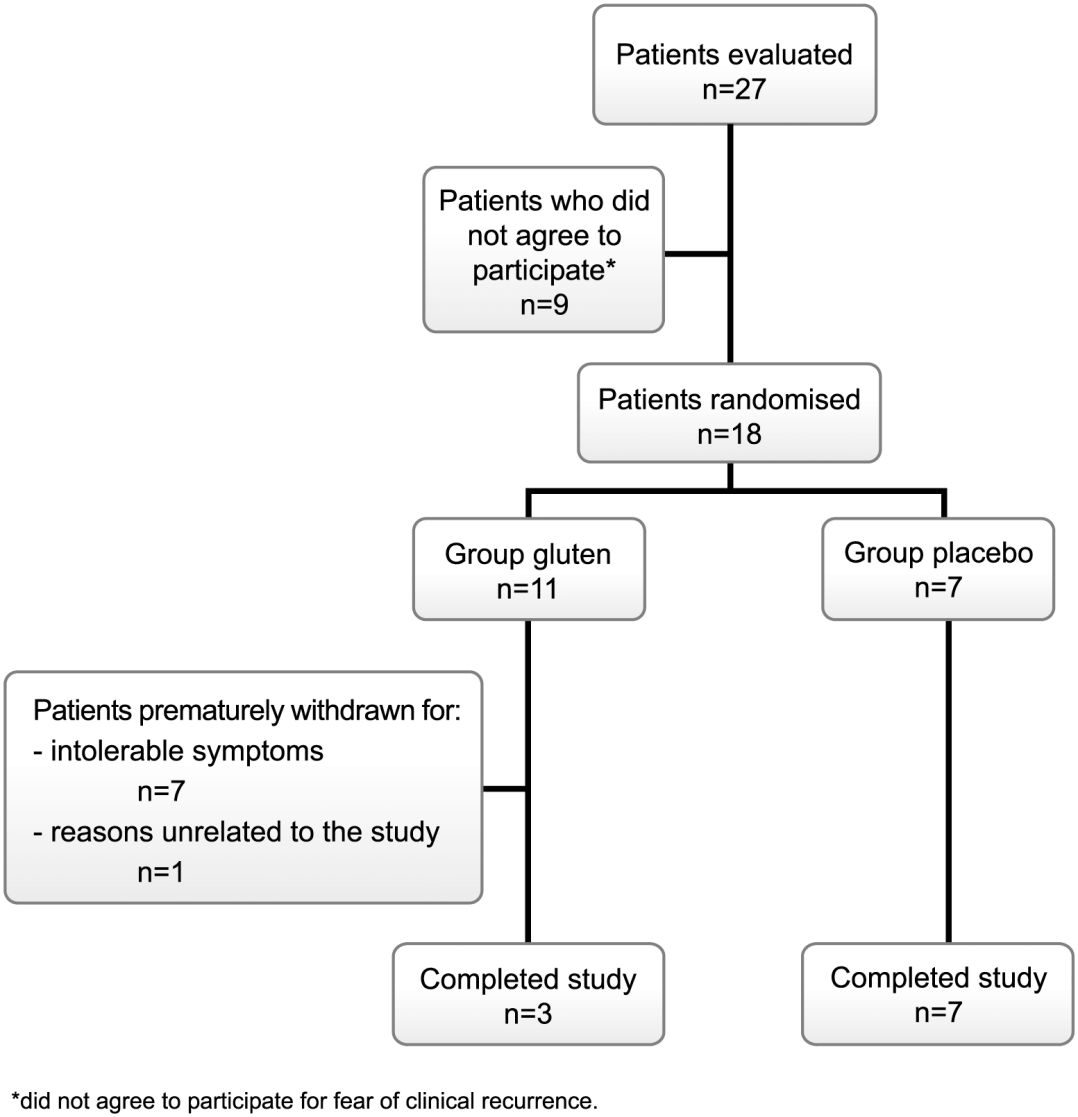
Flow of patient recruitment and reasons for screening failure and withdrawals.

Twenty-seven patients fulfilling inclusion criteria were evaluated. Nine patients did not agree to participate for fear of clinical recurrence. In all, 18 patients were randomised (gluten, 11; placebo, 7) and eligible for ITT analysis. The flow of patients throughout the study is represented in Fig 1. The baseline demographic and clinical characteristics were similar for the two treatment groups (Table 1). There was a trend toward a higher baseline clinical score in the gluten than the placebo group, but symptoms were mild (mean score, 120 *vs* 67, for a score ranging from 0 to 400), and there were no differences in HRQoL. S1 Fig presents the evolution of IEL count after GFD, previous to trial inclusion. Adherence to GFD was excellent throughout the study; in addition, adherence with the gluten/placebo sachets was also excellent, above 90% in all patients and without differences between study groups.

**Table 1:** Baseline characteristics of the randomised patients.

	*GLUTEN n = 11*	*PLACEBO n = 7*	*P value*
Age (years)	49.5±3.3	53.2±4.8	0.52
Sex (M/F)	1/10	1/6	0.73
Clinical symptoms at presentation*			
Diarrhoea	5/11	3/7	0.65
Abdominal bloating + flatulence	7/11	4/7	0.58
Iron-deficiency anaemia	0/11	2/7	0.14
Concomitant autoimmune disease	1/11	2/7	0.53
IEL at diagnosis (median, range)	35 (31–40)	32 (31–45.5)	0.92
IEL at follow-up with GFD (median, range)	16 (12–22)	20 (17.5–23)	0.34
IEL at inclusion (median, range)	22 (19–26)	25 (22–39)	0.17
Coeliac cytometry at inclusion (%)	5 (45.4%)	2 (29%)	0.64
Anti-tTG2 intestinal deposits at inclusion (%)	2 (18%)	3 (43%)	0.32
Baseline clinical score (mean±SEM)	120±28	67±20	0.19
Baseline GIQLI (mean±SEM)	140.2±5.7	152±5.3	0.17

*All patients had GI symptoms at presentation.

IEL, intraepithelial lymphocytes (normal values, <25 per 100 epithelial cells, H&E count).

GIQLI, Gastrointestinal Quality of Life Index.

### Clinical evolution

**Fig 2:**
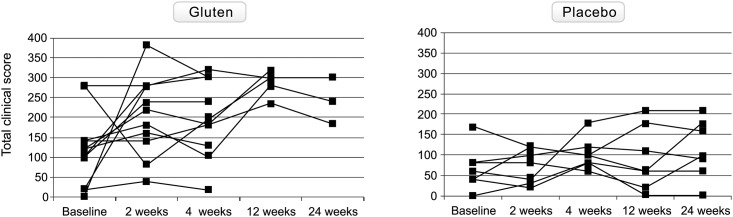
Evolution of the total clinical score throughout the study in individual patients of both gluten-treated and placebo groups.

The evolution of the total clinical score throughout the study in both groups is shown in Fig 2. Ten out of 11 (91%; 95% CI, 62–98%) patients developed exacerbation of symptoms in response to gluten and did so within the first two weeks; 7 of them (63.6%) were prematurely withdrawn because of intolerable symptoms. The non-relapsing patient was also prematurely withdrawn after 4 weeks for reasons unrelated to the study.

In contrast, 2 out of 7 patients (28.5%; 95% CI, 8–64%; p = 0.012 *vs* gluten) developed exacerbation of symptoms with placebo, but none of them (0%) presented severe symptoms requiring premature withdrawal (p<0.01 *vs* gluten).

**Fig 3:**
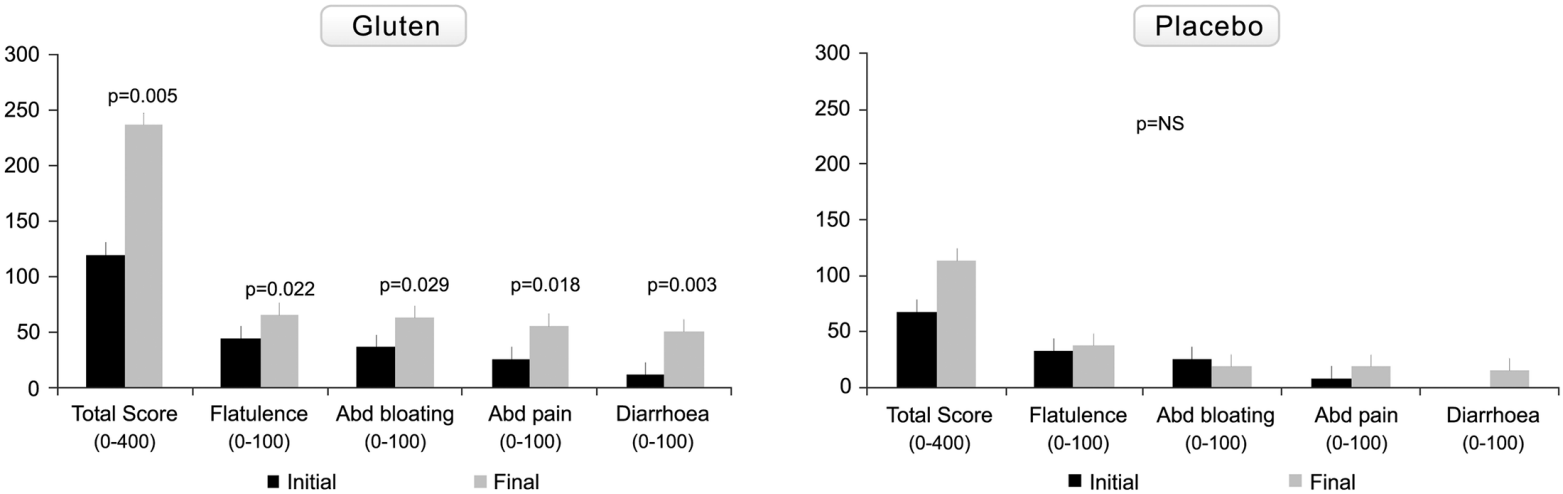
Changes in clinical symptom visual analogue scales from baseline to the end of the study in the gluten-treated and placebo groups. Total score represents the sum of values of the VAS of the different clinical symptoms. Results are expressed as mean±SEM.

As shown in Fig 3, representing changes in symptom scales (according to VAS) from baseline to the end of the study, the scores for overall symptoms, flatulence, abdominal bloating, abdominal pain, and diarrhoea worsened significantly only after gluten challenge. Serum anti-tTG2 and EmA antibodies remained negative in all patients.

### Health related quality of life

**Fig 4:**
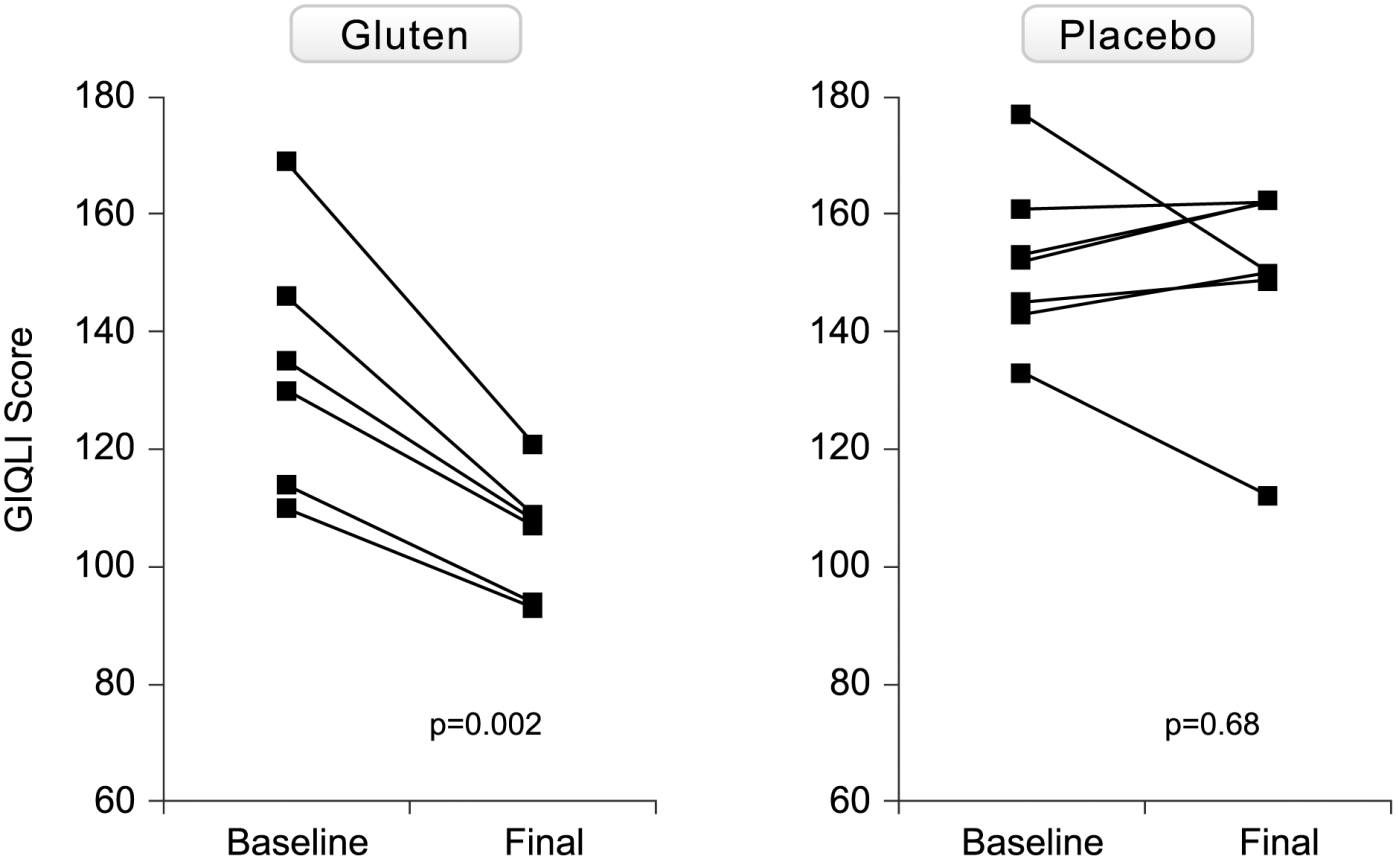
Evolution of health-related quality of life as assessed with the GIQLI in both gluten-treated and placebo groups.

The evolution of HRQoL assessed by the GIQLI in both groups is shown in Fig 4. The five patients prematurely withdrawing in the first 2 weeks after gluten challenge did not complete the questionnaire at withdrawal. In the other 6, there was a significant deterioration of HRQoL. There was no significant change in HRQoL with placebo.

In the gluten group, there was significant worsening in the symptom, physical and social scales, from baseline to end of the study (S2 Fig). There were no statistically significant differences in any of the scales evaluated from baseline to end of the study in the placebo group.

### Histology and tissue CoD markers

The evolution of IEL from baseline to final visit in both groups is shown in Table 2. Seven out of 11 patients in the gluten group had a follow-up biopsy after a median of 12 weeks (IQ range, 4 to 24) of challenge. No patient developed atrophy. There were no significant differences in IEL count when comparing baseline to final. However, there was an increase in IEL count in 3 of the 7 patients, all 3 showing increase in CD3+γδ+ IEL (two of them had received the gluten challenge during 6 months, and the other was withdrawn at 3 months).

**Table 2:** The evolution of IEL count, CD3+γδ+ and IgA tTG deposits from baseline to final visit in the placebo and gluten-treated groups.

Group	IEL		CD3+γδ+		IgA tTG deposits	
	Baseline	Final	Baseline	Final	Baseline	Final
placebo	25	23	12	7.3	neg	neg
placebo	29	15	34.5	38.8	neg	neg
placebo	22	21	3.6	5.3	+	neg
placebo	57	41	1.9	1.3	neg	+
placebo	23	26	2.4	4.8	++	neg
placebo	20	95	7.1	1.5	neg	neg
placebo	40	29.6	5.5	3.4	+	++
gluten	19	15.5	6.36	4.27	neg	neg
gluten	17	ND	2.71	ND	neg	ND
gluten	26	18	11.57	9.43	neg	neg
gluten	23	31	8.55	9.43	++	neg
gluten	28	34	41.46	36.4	+	++
gluten	20	23.5	1.6	2.6	neg	neg
gluten	13	ND	6.09	ND	neg	ND
gluten	42	ND	1.39	ND	neg	ND
gluten	21	20	8.68	8.5	neg	neg
gluten	31	ND	8.16	ND	neg	ND
gluten	7.5	15.8	15.18	20.75	neg	++

^+^, low intensity tTG deposits;

^++^, high intensity tTG deposits (see S2 File).

IEL, intraepithelial lymphocytes assessed by standard histological H&E count (normal values, <25 per 100 epithelial cells); CD3+γδ+, assessed by flow cytometry (normal values <8.5%).

ND, not done.

At baseline, 5 out of 11 patients in the gluten group had a coeliac IEL cytometric pattern, and 2 of them also had IgA tTG deposits. After gluten challenge, the same 5 patients had increased CD3+γδ+ IEL, while IgA tTG deposits persisted in one of them and appeared *de novo* in another.

The presence of tissue CoD markers in the baseline biopsies allowed classifying 5 out of 11 patients of the gluten challenge group as probable ‘coeliac lite’ disease cases (45.5%). The clinical worsening after the gluten challenge in these 5 patients supports this diagnosis.

The 7 patients in the placebo group had a follow-up biopsy at 24 weeks (Table 2). There were no significant differences in IEL count when comparing baseline to final. Two out of 7 patients had increased CD3+γδ+ IEL at baseline, and 3 additional patients had IgA tTG deposits. At follow-up biopsies (all at 24 weeks), after a strict GFD supervised by a dietician, the increase in CD3+γδ+ IEL persisted in only 1 patient and tTG deposits persisted in another. In 1 additional patient, IgA tTG deposits appeared *de novo*.

## Discussion

Since Michael N Marsh’s original descriptions, Marsh grade 1 lesion has been considered as part of the spectrum of CoD [7]. In recent years it has been reported that about 40% of NCGS patients have an increase in IEL count up to 40%. Therefore, it becomes important to make a proper differential diagnosis between CoD and NCGS in patients showing a Marsh 1 lesion. In the former, GFD probably should be as strict as it is in patients with atrophy, whereas in the latter the objective is to achieve relief of symptoms. In this sense, positive coeliac serology clearly supports a CoD diagnosis in these patients [24].

Recent trials suggest that gluten challenge induces symptom recurrence in only a minority of patients who meet clinical criteria for NCGS [17,18], suggesting that other components of wheat might be the triggers of symptoms in NCGS. In contrast to these studies, the present trial demonstrates that there is a subgroup of patients fulfilling the criteria for NCGS in whom gluten-challenge was associated with clinical relapse (in 90% of patients), whereas this only occurred in 28% of patients challenged with placebo. Since these patients were characterized by gastro-intestinal clinical symptoms within the clinical spectrum of CoD, presence of HLA-DQ2/8+, Marsh stage 1 lesion, and a clinical and histological response to a GFD, the question remains as to whether this condition should be considered a low-grade CoD (also called ‘coeliac lite’ by some authors) or NCGS.

Noteworthy, in spite of a relatively high daily gluten intake no patients developed positive serum IgA anti-tTG2 and none of those with follow-up biopsies developed atrophy, which are criteria previously used to consider CoD in these patients [25]. In this regard, a high γδ IEL count and/or the presence of anti-TG2 deposits in the mucosa have been thought to increase the likelihood of CoD, since these tissue markers appear to be extremely specific for CoD [8,23]. A recent study suggested that γδ IEL count using flow cytometry is more accurate than anti-TG2 deposits in detecting CoD in patients with Marsh 1 lesions and positive coeliac serology. Of note, the presence of an increase in γδ IEL count was 91% specific for CoD [23]. In the present study, these tissue coeliac markers were present in around 55% of patients at inclusion, despite their being on a GFD, suggesting a ‘coeliac lite’ disease. Previous studies of CoD with atrophy have shown a permanent increase in γδ IEL, even after a GFD [26], considering that this marker may provide a clue for CoD diagnosis and offering the possibility of identifying CoD patients when they are on a GFD, even when histological examination of the biopsy shows recovered mucosa. The results of the present study suggest that this assertion might also be applicable to Marsh 1 seronegative patients. Further studies are needed to ascertain the accuracy of this parameter as a CoD biomarker. Gluten challenge may be unnecessary in these patients, since the presence of an increase in γδ IEL might be of help to classify them as CoD. In contrast to CoD, the presence of increased T-cell receptor γδ IELs in NCGS is controversial, with one study showing increase [27] and another not [20]. In the former study performed in Finland, CoD was ruled out on the basis of a negative HLA-DQ2/8 study. However, the frequency of HLA-DQ2/8 negative CoD in Finland is around 2.4% [28], and thus CoD could not be definitely excluded only on the basis of negative celiac-type HLA.

Likewise, it is important to note that the presence of tTG deposits was less regular than γδ IEL+, probably reflecting both their non-homogeneous tissue distribution which could induce a biopsy sampling error, and their presence at low intensity in around 20% of healthy controls [23,29,30], which could yield false-positive interpretations.

The amount of gluten chosen for rechallenge merits some comment. The relatively high daily gluten intake used could be responsible for early symptoms and withdrawal. However, a dose of 20 g per day is similar to the amount used in a well-designed trial in NCGS patients [16]. In this trial 16 g/d of gluten did not trigger more symptoms than placebo. We used 20 g/d, which is the average consumed daily in Spain, because we aimed to demonstrate in a ‘proof-of-concept’ trial that gluten was responsible for symptoms in the particular subgroup of patients included in the present study.

It is still unclear whether NCGS is a permanent or transient condition [10,31]. In the present study, one patient (9%) with a previous clinical and histological improvement after GFD had no clinical relapse after gluten challenge and did not express tissue CoD markers at baseline, suggesting the existence of a transient NCGS. In this sense, a gluten challenge after 1–2 years on GFD has been advised [29], and our results confirm that this approach could be appropriate.

The present study has some limitations. We selected a parallel design since in previous studies using a cross-over design, an important nocebo response was observed. Regrettably, the present design precluded knowing what the final diagnosis of patients in the placebo arm was. However, the baseline presence of tissue CoD markers suggests that at least 4 out of the 7 patients could have a ‘coeliac lite’ disease. Another limitation was the small sample size analysed, in part due to a considerable number of patients not providing consent to participate in the trial because of fear of relapse. Also, in our unit CoD markers have been analysed in recent years at diagnosis and thus we have no more naïve patients for these measurements. In spite of this, the sample size was sufficient to demonstrate the main aim of the study, i.e., that most patients had gluten-dependent symptoms. In addition, the secondary aim was also confirmed, i.e., that in spite of patients being on a GFD, CoD markers were positive at baseline in more than a half of them.

In conclusion, this proof-of-concept study indicates that gluten is the trigger of symptoms in a subgroup of patients fulfilling the diagnostic criteria for NCGS, which were characterized by positive coeliac genetics, lymphocytic enteritis, and clinical and histological remission after a gluten-free diet.

## Supporting Information

S1 CONSORT Checklist CONSORT 2010 checklist of information to include when reporting a randomized trial. (DOCX)

S1 Fig Evolution of IEL histological count on GFD after initial diagnosis and prior to inclusion in the present trial. (TIF)

S2 Fig Changes in GIQLI scales from baseline to end of the study in the gluten and placebo-treated groups. Results are expressed as mean±SEM.
(TIF)

S1 File Gluten vs placebo (SPSS database). (SAV)

S2 File Supplementary methods. (DOCX)

S1 Protocol Protocol gluten vs placebo July 2011, English. (DOC)

S2 Protocol Protocol gluten vs placebo July 2011, Spanish. (DOC)

## Abbreviations

LE: lymphocytic enteritis
CoD: coeliac disease
NCGS: non-coeliac gluten sensitivity
FODMAPs: fermentable oligo-, di-, monosaccharides and polyols
GFD: gluten-free diet
Anti-TG2: anti-tissue transglutaminase antibodies
EmA: anti-endomysium antibodies
VAS: visual analogue scale
GIQLI: Gastrointestinal Quality of Life Index
HRQoL: Health-Related Quality of Life
IEL: intraepithelial lymphocytes

## References

[1] Hayat M, Cairns A, Dixon MF, O'Mahony S. Quantitation of intraepithelial lymphocytes in human duodenum: what is normal? J Clin Pathol 2002;55:393–4. 11986350 10.1136/jcp.55.5.393 PMC1769642

[2] Mahadeva S, Wyatt JI, Howdle PD. Is a raised intraepithelial lymphocyte count with normal duodenal villous architecture clinically relevant? J Clin Pathol 2002;55:424–8. 12037023 10.1136/jcp.55.6.424 PMC1769667

[3] Brown I, Mino-Kenudson M, Deshpande V, Lauwers GI. Intraepithelial lymphocytosis in architecturally preserved proximal small intestinal mucosa: an increasing diagnostic problem with a wide differential diagnosis. Arch Pathol Lab Med 2006;130:1020–5. 16831028 10.5858/2006-130-1020-ILIAPP

[4] Rosinach M, Esteve M, González C, Temiño R, Mariné M, Monzón H, et al Lymphocytic duodenosis: aetiology and long-term response to specific treatment. Dig Liver Dis 2012;44:643–8. 10.1016/j.dld.2012.03.006 22497904

[5] Aziz I, Evans KE, Hopper AD, Smillie DM, Sanders DS. A prospective study into the aetiology of lymphocytic duodenosis. Aliment Pharmacol Ther 2010;32:1392–7. 10.1111/j.1365-2036.2010.04477.x 21050242

[6] Santolaria S, Dominguez M, Alcedo J, Abascal M, García-Prats MD, Marigil M, et al Lymphocytic duodenosis: etiological study and clinical presentations. Gastroenterol Hepatol 2013;36:565–73. 10.1016/j.gastrohep.2013.06.003 24007857

[7] Rostami K, Aldulaimi D, Holmes G, Johnson MVV, Robert M, Srivastava A, et al Microscopic enteritis: Bucharest consensus. World J Gastroenterol 2015;21:2593–604. 10.3748/wjg.v21.i9.2593 25759526 PMC4351208

[8] Husby S, Koletzko S, Korponay-Szabó IR, Mearin ML, Phillips A, Shamir R, et al European Society for Pediatric Gastroenterology, Hepatology, and Nutrition guidelines for the diagnosis of coeliac disease. J Pediatr Gastroenterol Nutr 2012;54:136–60. 10.1097/MPG.0b013e31821a23d0 22197856

[9] Catassi C, Fasano A. Coeliac disease diagnosis: simple rules are better than complicated algorithms. Am J Med 2010;123:691–3. 10.1016/j.amjmed.2010.02.019 20670718

[10] Sapone A, Bai JC, Ciacci C, Dolinsek J, Green PH, Hadjivassiliou M, et al Spectrum of gluten-related disorders: consensus on new nomenclature and classification. BMC Med 2012;10:13 10.1186/1741-7015-10-13 22313950 PMC3292448

[11] Volta U, Caio G, Tovoli F, De Giorgio R. Non-coeliac gluten sensitivity: questions still to be answered despite increasing awareness. Cell Mol Immunol 2013;10:383–92. 10.1038/cmi.2013.28 23934026 PMC4003198

[12] Molina-Infante J, Santolaria S, Sanders DS, Fernández-Bañares F. Systematic review: noncoeliac gluten sensitivity. Aliment Pharmacol Ther 2015;41:807–20. 10.1111/apt.13155 25753138

[13] Ferch CC, Chey WD. Irritable bowel syndrome and gluten sensitivity without coeliac disease: separating the wheat from the chaff. Gastroenterology 2012;142:664–6. 10.1053/j.gastro.2012.01.020 22281277

[14] Husby S, Murray JA. Gluten sensitivity: coeliac lite versus coeliac like. J Pediatr 2014; 164:436–8 10.1016/j.jpeds.2013.11.024 24411520

[15] Biesiekierski JR, Newnham ED, Irving PM, Barrett JS, Haines M, Doecke JD, et al Gluten causes gastrointestinal symptoms in subjects without coeliac disease: a double-blind randomized placebo-controlled trial. Am J Gastroenterol 2011;106:508–14. 10.1038/ajg.2010.487 21224837

[16] Biesiekierski JR, Peters SL, Newnham ED, Rosella O, Muir JG, Gibson PR. No effects of gluten in patients with self-reported non-coeliac gluten sensitivity after dietary reduction of fermentable, poorly absorbed, short-chain carbohydrates. Gastroenterology 2013;145:320–8. 10.1053/j.gastro.2013.04.051 23648697

[17] Zanini B, Baschè R, Ferraresi A, Ricci C, Lanzarotto F, Marullo M, et al Randomised clinical study: gluten challenge induces symptom recurrence in only a minority of patients who meet clinical criteria for non-coeliac gluten sensitivity. Aliment Pharmacol Ther 2015;42:968–76. 10.1111/apt.13372 26310131

[18] Di Sabatino A, Volta U, Salvatore C, Biancheri P, Caio G, De Giorgio R, et al Small amounts of gluten in subjects with suspected noncoeliac gluten sensitivity: a randomized, double-blind, placebo-controlled, cross-over trial. Clin Gastroenterol Hepatol 2015;13:1604–12. 10.1016/j.cgh.2015.01.029 25701700

[19] Shahbazkhani B, Sadeghi A, Malekzadeh R, Khatavi F, Etemadi M, Kalantri E, et al Non-coeliac gluten sensitivity has narrowed the spectrum of irritable bowel syndrome: a double-blind randomized placebo-controlled trial. Nutrients 2015;7:4542–54. 10.3390/nu7064542 26056920 PMC4488801

[20] Sapone A, Lammers KM, Mazzarella G, Mikhailenko I, Cartenì M, Casolaro V, et al Differential mucosal IL-17 expression in two gliadin-induced disorders: gluten sensitivity and the autoimmune enteropathy coeliac disease. Int Arch Allergy Immunol 2010;152:75–80. 10.1159/000260087 PMC2956008 19940509

[21] Jaeschke R, Singer J, Guyatt GH. A comparison of seven-point and visual analogue scales. Data from a randomized trial. Control Clin Trials 1990;11:43–51. 2157581 10.1016/0197-2456(90)90031-v

[22] Quintana JM, Cabriada J, López de Tejada I, Varona M, Oribe V, Barrios B, et al Translation and validation of the gastrointestinal Quality of Life Index (GIQLI). Rev Esp Enferm Dig 2001;93:693–706. 11995369

[23] Fernández-Bañares F, Carrasco A, García-Puig R, Rosinach M, González C, Alsina M, et al Intestinal intraepithelial lymphocyte cytometric pattern is more accurate than subepithelial deposits of anti-tissue transglutaminase IgA for the diagnosis of coeliac disease in lymphocytic enteritis. PLoS One 2014;9:e101249 10.1371/journal.pone.0101249 25010214 PMC4091865

[24] Kurppa K, Collin P, Viljamaa M, Haimila K, Saavalainen P, Partanen J, et al Diagnosing mild enteropathy coeliac disease: a randomized, controlled clinical study. Gastroenterology 2009;136:816–23. 10.1053/j.gastro.2008.11.040 19111551

[25] Aziz I, Key T, Goodwin JG, Sanders DS. Predictors for celiac disease in adult cases of duodenal intraepithelial lymphocytosis. J Clin Gastroenterol 2015;49:477–82. 10.1097/MCG.0000000000000184 25014240

[26] Camarero C, Eiras P, Asensio A, Leon F, Olivares F, Escobar H, et al Intraepithelial lymphocytes and coeliac disease: permanent changes in CD3-/CD7+ and T cell receptor gammadelta subsets studied by flow cytometry. Acta Paediatr 2000;89:285–90. 10772275

[27] Kaukinen K, Turjanmaa K, Mäki M, Partanen J, Venäläinen R, Reunala T, et al Intolerance to cereals is not specific for coeliac disease. Scand J Gastroenterol 2000;35:942–6. 11063153 10.1080/003655200750022995

[28] Polvi A, Arranz E, Fernandez-Arquero M, Collin P, Mäki M, Sanz A, et al HLA-DQ2-negative celiac disease in Finland and Spain. Hum Immunol 1998;59:169–75. 9548076 10.1016/s0198-8859(98)00008-1

[29] Maglio M, Tosco A, Auricchio R, Paparo F, Colicchio B, Miele E, et al Intestinal deposits of anti-tissue transglutaminase IgA in childhood celiac disease. Dig Liver Dis 2011;43:604–8. 10.1016/j.dld.2011.01.015 21342796

[30] Koskinen O, Collin P, Lindfors K, Laurila K, Mäki M, Kaukinen K. Usefulness of small-bowel mucosal transglutaminase-2 specific autoantibody deposits in the diagnosis and follow-up of celiac disease. J Clin Gastroenterol 2010;44:483–82. 10.1097/MCG.0b013e3181b64557 19779364

[31] Volta U, Caio G, De Giorgio R, Henriksen C, Skodje G, Lundin KE. Non-coeliac gluten sensitivity: A work-in-progress entity in the spectrum of wheat-related disorders. Best Pract Res Clin Gastroenterol 2015;29:477–91. 10.1016/j.bpg.2015.04.006 26060112

