# Supplementary figures and images for: Double-Blind Randomized Clinical Trial: Gluten versus Placebo Rechallenge in Patients with Lymphocytic Enteritis and Suspected Celiac Disease
Source: PLoS One. 2016 Jul 8;11(7):e0157879. doi: 10.1371/journal.pone.0157879 (PMC4938236; doi:10.1371/journal.pone.0157879)

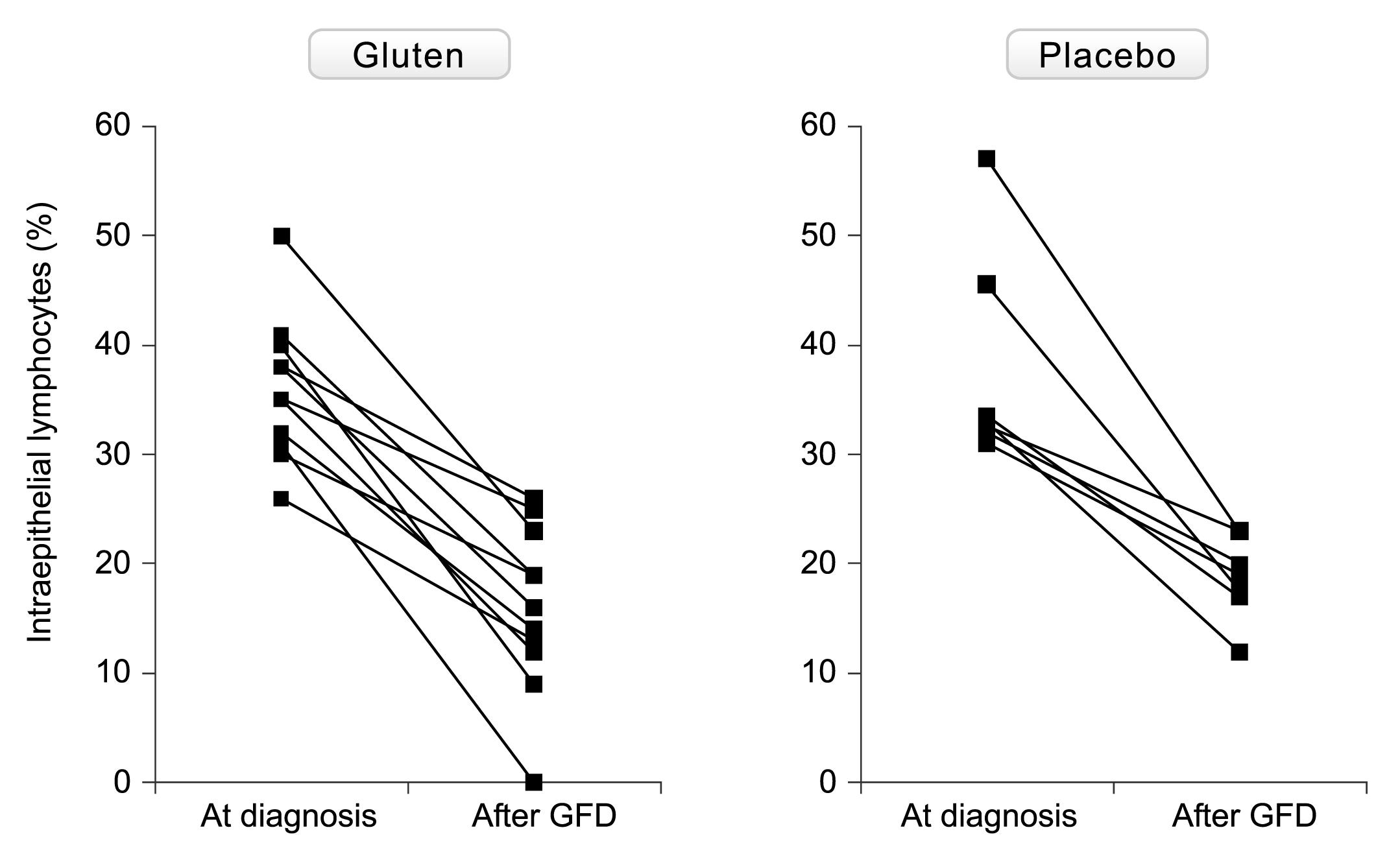

Supplement: S1 Fig — (TIF) [file pone.0157879.s002.tif]

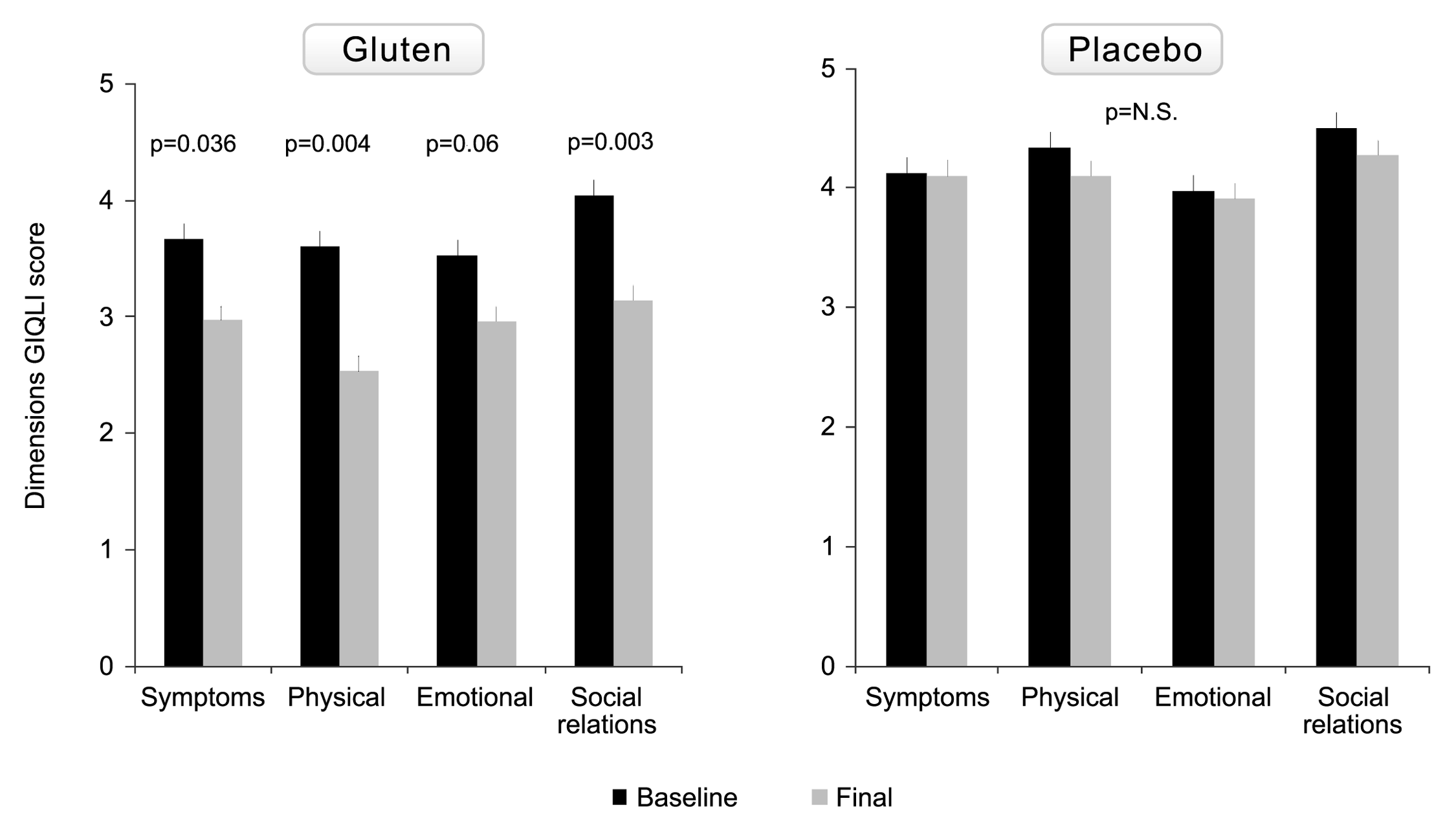

Supplement: S2 Fig — Results are expressed as mean±SEM. (TIF) [file pone.0157879.s003.tif]
