# Supplementary material for: Double-Blind Randomized Clinical Trial: Gluten versus Placebo Rechallenge in Patients with Lymphocytic Enteritis and Suspected Celiac Disease
Source: PLoS One. 2016 Jul 8;11(7):e0157879. doi: 10.1371/journal.pone.0157879 (PMC4938236; doi:10.1371/journal.pone.0157879)
Supplement: S1 Protocol — (DOC) [file pone.0157879.s006.doc]

# CLINICAL TRIAL PROTOCOL

**Lymphocytic enteritis with negative coeliac serology and clinical and histological response to a gluten-free diet: double-blind randomized clinical trial with rechallenge of gluten vs. placebo.**

## Phase IV trial

**Code: EL-DSG/07/2011**

### Version: June 2011

### AGREEMENT SHEET

**______________________________________________________________________**

**Protocol title:**

Lymphocytic enteritis with negative coeliac serology and clinical and histological response to a gluten-free diet: double-blind randomized clinical trial with rechallenge of gluten vs. placebo.

**Sponsor:**

Digestive System Service

Mutua Terrassa University Hospital

Plaza Dr Robert, 5

08221 Terrassa (Barcelona)

Spain

**Project leader:**

Dr. Fernando Fernández Bañares

Adjunct physician, Digestive System Service

Mutua Terrassa University Hospital

Date

Signature

Date of first draft: February 2011

Date of final version: June 2011

# 1. SUMMARY OF THE STUDY

| **Title** | Lymphocytic enteritis with negative coeliac serology and clinical and histological response to a gluten-free diet: double-blind randomized clinical trial with rechallenge of gluten vs. placebo. | |
| --- | --- | --- |
| **Study code** | EL-DSG/07/2011 | |
| **Objectives** | To demonstrate hypersensitivity to gluten in patients with lymphocytic enteritis, and clinical response and histology of a gluten-free diet despite the presence of IgA negative tissue anti-transglutaminase antibodies. | |
| **Treatment** | Sachets with gluten (10 g) or placebo (maltodextrin: 10 g): 1 sachet every 12 hours. | |
| **Study design** | Prospective, randomized, double-blind, placebo-controlled, with parallel groups of gluten rechallenge vs. placebo, for 24 weeks, in patients following a gluten-free diet.   - Group A: Powdered gluten (10 g every 12 hours) - Group B: Placebo (10 g every 12 hours)   Determination of digestive clinical state, adherence to diet, coeliac serology, histology, flow cytometry, and tissue anti-transglutaminase antibodies in intestinal mucus membrane, at baseline and after gluten rechallenge or placebo (whether clinical digestive symptoms reappear at 4 or 12 weeks, or at the conclusion of the study at 24 weeks). | |
| **Study population** | Inclusion criteria:  1. Signed informed consent  2. Age 18 or older  3. Confirmed histological diagnosis of lymphocytic enteropathy (LE)  5. Coeliac-positive genetic study (HLA-DQ2 and/or HLA-DQ8)  6. Negative coeliac serology  7. Complete clinical and histological response to gluten-free diet  8. Initial clinical signs with or without extra-intestinal manifestations | |
|  | Exclusion criteria:  1. Inability to adhere to the scheduled visits or other protocol requirements, in the judgement of the investigator  2. Participation in a clinical trial in the previous 30 days, simultaneous participation in a trial, or prior participation in the present study  3. History of gluten-sensitive enteropathy with villous atrophy or positive serology  4. Lymphocytic enteropathy or initial response to gluten-free diet but following a normal diet at the time of selection | |
| **Sample size** | 20 patients | |
| **Length of treatment** | 24 weeks | |
| **Primary efficacy variables** | Reappearance of clinical digestive signs and histological recurrence | |
| **Secondary efficacy variables** | - Clinical signs at 4 weeks - Clinical signs at 12 weeks - Flow cytometry to measure percentage of lymphocytes CD3+ gamma-delta+ and CD3- in intestinal mucus membrane | - Assessment of IgA tissue anti-transglutaminase antibodies in the intestinal mucus membrane - IgA tissue anti-transglutaminase antibodies in serum and anti-endomysium antibodies |

**2. TABLE OF CONTENTS**

1. Summary of the study 3
2. Table of contents 5
3. General information 6
4. Justification and objectives 7
5. Type and design of clinical trial 8
6. Subject selection 9
7. Description of treatment 10
8. Development of trial and assessment of response 11
9. Adverse events 13
10. Ethical considerations 18
11. Accountability 20
12. Additional factors 22
13. Statistical analysis 23
14. References 24

Appendix 1. Gluten 10 g single-dose product characteristics 26

Appendix 2. Maltodextrin 10 g single-dose product characteristics 28

Appendix 3. Patient quality of life 30

Appendix 4. Three-day diet log 33

Appendix 5. Gluten-free diet compliance assessment 43

Appendix 6. Patient global symptom assessment 44

Appendix 7. Patient information sheet 45

Appendix 8. Informed consent form 49

**3. GENERAL INFORMATION**

**3.1. CODE**

EL-DSG/07/2011

**3.2. TITLE**

Lymphocytic enteritis with negative coeliac serology and clinical response and histology with a gluten-free diet: double-blind randomized clinical trial with rechallenge of gluten vs. placebo.

**3.3. TYPE OF TRIAL**

Phase IV clinical trial.

**3.4. DESCRIPTION OF THE STUDY THERAPY**

Gluten is a protein found in cereals such as wheat, oats, rye, and barley, made up of gliadins and glutenins. It is therefore widely present in foods. The average daily intake of gluten is 20 grams.

Gluten will be administered in 10-gram sachets.

**3.5. SPONSOR**

Digestive System Service

Mutua Terrassa University Hospital

Plaza Dr Robert, 5

08221 Terrassa (Barcelona), Spain

**3.6. TECHNICAL DIRECTOR RESPONSIBLE FOR SAMPLE PREPARATION**

Jaume Llorente

Xalabarder Pharmacy

Terrassa

**3.7. IDENTIFICATION OF THE QUALITY GUARANTEE MONITOR**

Maria Esteve Comas

Gastroenterologist

Digestive System Service

Mutua Terrassa University Hospital

**3.8. EXPECTED DURATION OF TRIAL**

Maximum recruitment period: 30 months

Maximum treatment period: 6 months

Maximum total duration: 36 months

**4. JUSTIFICATION AND OBJECTIVES**

**4.1. JUSTIFICATION**

In recent years the concept of coeliac disease (CD) has undergone a profound re-examination. A wide range of clinical manifestations and histological presentations has been recognized, and significant progress has been made in learning about the genetic and immunological mechanisms that underlie the disease. The common denominator for all CD patients is the presence of a variable combination of clinical manifestations that are gluten-dependent and antibody-specific (tissue anti-transglutaminase 2, anti-endomysium), with the haplotypes HLA-DQ2 and/or DQ8 and various degrees of enteropathy ranging from epithelial lymphocyte infiltration (lymphocytic enteropathy, LE) to complete villous atrophy (1).

In addition, there is another condition known as non-coeliac gluten sensitivity (NCGS), the exact nature and limits of which, and the degree to which it may overlap with CD, are as yet poorly understood (2, 3). At present NCGS takes in a series of morphological, functional, and immunological alterations occurring in the absence of any of the key characteristics of CD (enteropathy, associate HLA haplotypes, and the presence of anti-tTG2 antibodies) but with a clinical response to the exclusion of dietary gluten (4). Along this line, patients have been described with NCGS without enteropathy (Marsh 0). Other patients have been described with only minimal changes in the mucus membrane—that is, with enteropathy (LE, Marsh 1). Unlike CD, NCGS is not associated with a genetic predisposition; it may be reversible, and the gluten-free diet for it needn’t be as strict as that for CD. In recent years, many patients have been diagnosed in our service with enteropathy that is sensitive to gluten, within the CD spectrum, on the basis of the presence of LE, positive coeliac genetic study, and clinical and histological response to a gluten-free diet, despite their having negative specific serology. On the basis of current understanding we wondered whether these patients presented CD, or whether some of them may in fact have an NCGS. Being able to assess the clinical and histological response to the reintroduction of gluten, as well as being afforded the chance to make specific immunological determinations in the intestinal mucosa, would allow us to make a definitive diagnosis of CD in these patients. Adhering to a gluten-free diet for life is no easy matter; for this reason alone a diagnosis of CD should be based on definitive evidence. Therefore, the results of the present study should go a long way toward broadening the spectrum of what today is called CD, and should also be of wide applicability in normal clinical practice.

1. Di Sabatino A, Corazza GR. Coeliac disease. Lancet 2009; 373:1480-93.
2. Troncone R, Jabri B. Coeliac disease and gluten sensitivity. J Intern Med 2011;269:582-90.
3. Bizzaro N, Tozzoli R, Villalta D Fabris M, Tonutti E. Cutting-edge issues in celiac disease and in gluten intolerance. Clinic Rev Allerg Immunol 2010; Dec 23.
4. Biesiekierski JR, Newnham de, Irving PM, Barrett JS, et al. Gluten causes gastrointestinal symptoms in subjects without celiac disease: A double-blind randomized placebo-controlled trial. Am J Gastroenterol 2011 (online only, 11 January 2011).

**4.2. OBJECTIVES**

To demonstrate hypersensitivity to gluten in patients with lymphocytic enteropathy and a clinical and histological response to a gluten-free diet in spite of presenting IgA negative tissue anti-transglutaminase antibodies.

**5. TYPE AND DESIGN OF CLINICAL TRIAL**

This is a phase IV double-blind clinical trial, controlled with placebo in parallel and with random assignment of treatment type.

**5.1. RANDOMIZATION**

A 1:1 proportion will be followed in making random assignment of participants to treatment groups. Assignment will be made using a computer, and medication coded consecutively with numbers for dispensation, following a double-blind schema, in the hospital pharmacy at the admission of each subject to the study.

On day 1 of the study each randomized patient will receive a participant number which will also be used for his or her medication. This randomized number will be associated with a specific treatment.

**5.2. MASKING METHODS**

In order to mask the gluten and placebo supplements they will be given the same organoleptic characteristics. They will be administered in non-transparent sachets filled with dry powder, with no difference in appearance between the consecutively numbered product packaging for the two treatment groups.

**6. SUBJECT SELECTION**

**6.1. INCLUSION CRITERIA**

1. Signed informed consent

2. Age 18 or older

3. Confirmed histological diagnosis of lymphocytic enteropathy (LE)*

5. Coeliac-positive genetic study (HLA-DQ2 and/or HLA-DQ8)

6. Negative coeliac serology

7. Complete clinical and histological response to gluten-free diet

8. Initial clinical signs with or without extra-intestinal manifestations

*Lymphocytic enteropathy is defined as an intraepithelial lymphocyte count greater than 25 per 100 epithelial cells, using histochemical techniques with anti-CD3 monoclonal antibodies for the count.

**6.2. EXCLUSION CRITERIA**

1. Inability to adhere to the scheduled visits or other protocol requirements, in the judgement of the investigator

2. Participation in a clinical trial in the previous 30 days, simultaneous participation in a trial, or prior participation in the present study

3. History of gluten-sensitive enteropathy with villous atrophy or positive serology

4. Lymphocytic enteropathy or initial response to gluten-free diet but following a normal diet at the time of selection

**6.3. EXPECTED NUMBER OF SUBJECTS**

The estimated sample size is 20 patients (10 for each treatment group) who will be evaluated on the basis of intention to treat.

**7. DESCRIPTION OF TREATMENT**

The subjects included in the study will be randomly distributed to two therapeutic groups. The randomly assigned code of each will determine which of the two treatment options is followed:

**Group 1 (Group without gluten)**: Will follow a gluten-free diet and will receive placebo as supplement (10 grams of powdered maltodextrin; Appendix 2), 1 sachet every 12 hours, mixed with food (puree, soup).

**Group 2 (Group with gluten)**: Will follow a gluten-free diet and will receive gluten as supplement (10 grams of powdered gluten; Appendix 1), 1 sachet every 12 hours, mixed with food (puree, soup).

**7.1. CONCOMITANT TREATMENT**

Any medication that needs to be taken will be noted on the page for this purpose in the data collection documentation.

**7.2. MEANS TO ASSESS COMPLIANCE**

Assessment of compliance will be made by counting the number of sachets returned at each visit and by reviewing what is recorded in the patient diary.

A standard gluten-free diet will be prescribed in writing. A dietician or expert nutritionist will see each patient each month to evaluate how closely the diet is being followed (Appendices 4 and 5).

**7.3. GUIDELINES FOR MANAGEMENT OF STUDY MEDICATION**

The distribution of the medication samples for the clinical trial will be made through the pharmacy service. The pharmacist/investigator will complete, and return to the sponsor, the sheets confirming receipt of the sachets of therapeutic material; they will be responsible for the proper handling and storage of the medication, and for that which is left over at the conclusion of the study. All medication used in the study must be stored in a secure location throughout the trial.

All of the medication will be carefully accounted for. At the conclusion of the study a copy of the inventory will be filed by the investigator. Leftover medication will be returned to the sponsor’s pharmacy service for destruction.

**7.4. PACKAGING AND LABELING**

The medication for the trial will be distributed to subjects in the form of sachets, either of gluten or maltodextrin according to the randomization status of the participant. The sachets will be numbered in correlated order and will be assigned in rigorous order in accordance with the inclusion of subjects in the study. The evaluator will at all times be blind to the randomization key.

**8. DEVELOPMENT OF TRIAL AND ASSESSMENT OF RESPONSE**

**8.1. PRINCIPAL AND SECONDARY ASSESSMENT VARIABLES**

**Efficacy criteria**

- Primary efficacy evaluation variable: Reappearance of clinical digestive signs (24 weeks)
- In addition, secondary efficacy variables will be evaluated:
  - Clinical signs at 4 weeks
  - Clinical signs at 12 weeks
  - Flow cytometry to measure percentage of lymphocytes CD3+ gamma-delta+ and CD3- in intestinal mucus membrane
  - Assessment of IgA tissue anti-transglutaminase antibodies in the intestinal mucus membrane
  - IgA tissue anti-transglutaminase antibodies in serum and anti-endomysium antibodies
  - Quality of life according to the GIQLI questionnaire.
  - Histological changes on biopsy in the distal duodenum

**Safety criteria**

The gluten-free diet in itself is entirely free of side effects. Nevertheless, a registry of typical side effects will be maintained throughout the study.

**8.2. DEVELOPMENT OF TRIAL, NUMBER OF VISITS, COMPLEMENTARY EXPLORATIONS**

The subjects will be selected according to the inclusion and exclusion criteria established in this protocol. In the event of a patient fulfilling the inclusion criteria and none of the exclusion criteria, he or she will be informed of the existence of the study, of its characteristics, and of the advantages and disadvantages in participating in it. Patients will be given an information sheet and their consent to participate in the study will be requested.

Those patients agreeing to take part in the study will receive a numerical code with which they will randomly receive one or the other of the medical treatment types.

After the start-up, the subject will be seen at 1, 3, and 6 months, as stipulated, and more frequently if necessary.

In the pre-inclusion visits, comprehensive bloodwork, coeliac serology, coeliac genetic study (HLA-DQ2/HLA-DQ8, if not already done), and distal duodenal biopsy, for pathological anatomy and flow cytometry, will be carried out.

**8.3. DESCRIPTION OF METHODS USED TO ASSESS RESPONSE**

Clinical response will be measured with analogue visual scales ranging from 0 to 100, administered at baseline and at each visit up to 6 months, for symptoms of abdominal pain, abdominal swelling, diarrhea, and flatulence (total score 0 to 400 points) (Appendix 6).

Patient quality of life will be evaluated in the first visit and the last (at 6 months, or at early withdrawal) using the GIQLI questionnaire (Gastrointestinal Quality of Life Index), which has been shown to yield valid, reliable scores for the detection of significant clinical changes in the lives of patients regarding their health (Eypasch E et al. Gastrointestinal Quality of Life Index: development, validation and application of a new instrument. Br J Surg 1995; 82:216-22; Quintana JM et al. Translation and validation of the Gastrointestinal Quality of Life Index (GIQLI). Rev Esp Enferm Dig 2001; 93:693-706) (See Appendix 3).

At the first visit and the last (or at early withdrawal from the study) a distal duodenal biopsy will be drawn for histological study, and evaluation of tTG deposits and lymphocytic sub-populations (using flow cytometry) will be made.

**8.4. WITHDRAWAL CRITERIA**

Any patient may withdraw from the study at any time without incurring any personal penalty and without the need for explanation. Withdrawal must be noted in the CRF when it occurs, along with the results to date and the reason(s) for withdrawal, if given.

The investigator may also withdraw a patient from the study upon consideration of the risks and benefits thereof, and determination that the former outweigh the latter with continued participation. The CRF must reflect the date and main cause for the withdrawal, as well as any further observations that may be made at the time of withdrawal. Among the reasons for withdrawing a patient early from the study are the following (the main reason should be indicated):

• Lack of efficacy of treatment, e.g.:

– Progression of the illness under study in comparison to baseline.

(in the six weeks following).

– The need to administer a concomitant medication that is prohibited in the treatment of the illness under study.

• Intolerable adverse events.

• Lack of patient cooperation, e.g.:

– Patient request.

– Lack of therapeutic compliance or failure to carry out visits according to the agreed calendar.

– Technical or logistical reasons (e.g., change of patient residence).

– Pregnancy or breast-feeding, either existing or planned.

• Others (must be noted), e.g.:

– Diagnosis different from that of the study.

All patients withdrawing early from the study must be seen within 7 days of the last administration of the medicine used in the study. This final visit is to be duly recorded in the CRF.

The investigator will continue to observe all patients withdrawing early from the study as a result of intolerable adverse events until the findings have been resolved.

**9. ADVERSE EVENTS**

**9.1.** **DEFINITIONS**

9.1.1. Adverse events

Adverse events (AE) are recorded at each visit in the patient notes (source document) and in the CRF.

An AE is any unfavorable medical incident affecting a patient or a clinical trial participant who has been given a pharmaceutical product. It is not necessarily causally related to the treatment. An AE may be, therefore, any sign (including an anomalous laboratory finding), symptom or illness, or accident co-occurring with the use of a medication, whether or not it is related to the medication. Examples include:

. A new diagnosis.

. A symptom requiring medical attention or leading to hospital admission of the patient

(surgery or accident).

. Any assumed adverse reaction to medication.

. Any symptom appearing in the patient’s notes.

. Any event related in time with the administration of the medication of the study and affecting the health of the patient (including changes in laboratory findings).

9.1.2. Serious adverse events

A serious adverse event (SAE) is any unfavorable medical incident that, at any dose:

. Causes death.

. Is potentially fatal.

. Warrants hospital admission of the patient or extension of a pre-existing hospitalization.

. Gives rise to a persistent or significant incapacity.

. Causes a congenital anomaly or birth defect.

Non-serious AEs are all those that do not correspond to any of the categories described immediately above.

9.1.3. Unexpected adverse drug reaction

An unexpected adverse drug reaction (ADR) is one in which the nature or severity of the reaction is not consistent with the available data concerning the product in question.

9.1.3.1. List of expected adverse drug reactions

No adverse events are expected in association with the administration of a gluten-free diet or with the gluten and placebo supplements in the present study.

**9.2.** **DOCUMENTATION AND NOTIFICATION**

9.2.1. Documentation and notification of adverse events

The patients must be instructed to contact the investigator immediately in the event of any incident that might require attention with appropriate measures.

In addition, during all the intermediate visits and the final visit the investigator should inquire about incidents, in general terms and without mentioning any symptom in particular. The question might take the following form: ‘Has your health worsened since the last time I saw you?’ If the answer is ‘no’ then no further questions are needed. But if the answer is ‘yes’ then the investigator should document the nature, time, severity, seriousness, and duration of the AE, as well as its cause.

For each AE the following data should be recorded:

. Nature of the event.

. When it began (date and time).

. Interval between the administration of the medication and the onset of the AE.

. Concomitant treatment: product (generic name, indication, dose, dosage interval, presentation, how administered, administration guidelines).

. Duration of AE

. Frequency

. Severity

. Seriousness

. Cause

. Measures taken

. Evolution

The severity of the event is to be evaluated in the following manner:

1. Mild: the patient notes the event or symptom, but tolerates it easily and it does not interfere with daily activities.
2. Moderate: the event or symptom interferes with daily activities.
3. Severe: the event or symptom prevents normal daily activities.

The relationship between an AE and the study medication is to be categorized in accordance with the WHO classification below (all the points need to be reasonably well met):

. Certain:

A clinical event, including a laboratory finding anomaly, the appearance of which is plausibly related to the administration of the medication and which may not be explained by the presence of a concurrent disease or the use of other drugs or chemical substances. The response to withdrawal of the medication (elimination of exposure) should include clinically plausible results. The event must be pharmacologically or phenomenologically definitive, to which end a satisfactory re-exposure procedure should be used, if necessary.

. Probable / presumable

A clinical event, including a laboratory finding anomaly, the appearance of which is sequentially related to the administration of the medication, and which is unlikely to be attributable to a concurrent disease or the use of other drugs or chemical substances and which presents a reasonable clinical response upon withdrawal of the medication (elimination of exposure). No information regarding re-exposure to the medication is needed to fulfill this definition.

. Possible

A clinical event, including a laboratory finding anomaly, the appearance of which is sequentially related to the administration of the medication, but which may also be explained by the presence of a concurrent disease or the use of other drugs or chemical substances. Information regarding the withdrawal of the medication may be non-existent or unclear.

. Improbable

A clinical event, including a laboratory finding anomaly, the temporal relation of which to the administration of the medication renders it unlikely that there is a causal relationship, and for which the use of other drugs or chemical substances or the existence of an underlying disease provide plausible explanations.

. Conditional / non-classified

A clinical event, including a laboratory finding anomaly, reported as an adverse reaction, for which appropriate evaluation requires additional data or for which the data are in the process of being analyzed.

. Non-evaluable / non-classifiable

A report in which an adverse reaction is recorded that cannot be evaluated because the information is insufficient or contradictory, and is not susceptible to being complemented or verified.

. Unrelated

There is enough available information to demonstrate that the etiology is unrelated to the medication in the study.

The measures adopted at the outset of adverse events are to be classified and described in the following manner:

1) None. That is, no changes were made in the study medication.

2) The medication was withdrawn from the study.

3) The adverse event or symptom was treated differently or medically.

4) Other measures (clear explanation).

The course run by the adverse event and its evolution are to be described in the following manner:

1) Recovered / resolved

2) Recovering / under resolution

3) Unrecovered / unresolved

4) Recovered / resolved with sequela(e)

5) Fatal

6) Unknown

Any patient suffering an AE is to receive follow-up after the conclusion of the study until the AE is resolved, or for a maximum of 4 weeks from the end of the patient’s participation in the study. Following the 4-week period, the investigator will issue a declaration concerning the evolution of the AE.

9.2.2. Documentation and notification of serious adverse events

At the moment when participants are included in the study they are to be instructed to contact the investigator in the event of a serious or unexpected AE, so that appropriate measures may be taken.

All SAEs (including death, independently of the cause thereof) occurring during the study or up to 14 days after its conclusion must be reported without delay—that is, within 24 hours—by fax to the study director, regardless of his or her involvement with the administration of the study medication. The minimum information required is the following: name of investigator/study center, patient’s name, patient’s initials, date of the initial dose, date of the last dose, date of the event, description of the event, evaluation of the causality, and the measures taken to counter the event.

Investigators will be provided with a specific form for reporting SAEs. In the event of an SAE, the physician must complete this form as an initial report and then submit it by fax to the project director. If necessary, a follow-up form must also be completed with any new information concerning the SAE; this updated SAE form is also to be submitted to the project director.

The report must contain a detailed description of the observed symptoms and the concomitant treatment given. In addition, the investigator must note whether there is a possible causal relation between the SAE and the medication used in the trial. He or she must carry out follow-up on each SAE until it is resolved or explained satisfactorily. The general procedure for observation must be applied to the fullest, with collection and analysis of the pharmacological risks (in matters of regulation) under the terms of the National Law on Medicines.

The sponsor may only unmask the treatment of isolated cases if it is a question of the safety of the study participant. When an intolerable SAE occurs, the patient will be withdrawn from the clinical trial, when the investigators deems this to be in order, and symptomatic treatment will then be administered. The measures taken are to be duly recorded in the CRF.

**10. ETHICAL CONSIDERATIONS**

This study will be carried out under the terms of the Helsinki Declaration (in its latest applicable version), international standards, and the applicable national regulations (see Section 10.3).

**10.1. INFORMATION FOR THE PATIENT AND PATIENT CONSENT**

Before their inclusion in this clinical trial, patients will be informed that participation is entirely voluntary and that they may withdraw at any time without the need to provide an explanation and without their doing so having any repercussion on their normal medical care.

Patients will be given information concerning the therapy of the study and its possible adverse effects. They will also be told about the aims of the study and its clinical significance. The explanation will also include information regarding insurance protection that they will be afforded and their obligations as insured parties.

The patients will be given ample time and opportunity to resolve any questions that they may have about the study. Furthermore, they will be given an information sheet containing all the essentials concerning the study, in writing. Appendices 7 and 8 represent the patient information sheet and the informed consent sheet.

Patient consent in writing must be obtained before beginning the study. By signing the informed consent, the patient declares that he or she is participating voluntarily in the trial with the intention of following the protocols that govern it and the instructions provided by the investigator, as well as being willing to answers questions posed during the course of the trial. Again, each patient will be reminded that participation in the study is entirely voluntary and that withdrawal from it may be made at any time without the need for any explanation. The investigator will keep a copy of the signed informed consent form of the patient in a location set aside for that purpose.

**10.2. ETHICAL COMMITTEE**

In line with the applicable national guidelines, before the beginning of the study this protocol and the patient informed consent and information sheets will be submitted to the ethical committee, for assessment under its governing criteria.

**10.3. INSURANCE POLICY**

The present study will undertake to investigate the effect of a gluten-free diet in comparison to a diet containing gluten (with the amount found in an average diet). A gluten-free diet is well tolerated, has all the macronutrients and micronutrients needed for proper nutrition, and is not associated with any side effects.

Nevertheless, an insurance policy will need to be taken out to cover civil liability.

**10.4. LEGAL CONSIDERATIONS**

The study must be carried out under the terms of the protocol, observing the Good Clinical Practice guidelines and the applicable regulations.

10.4.1. Helsinki Declaration

The study will be carried out in accordance with the ‘Ethical Principles for Medical Research Involving Human Subjects’ enshrined in the 18th General Assembly of the World Medical Association (WMA) in Helsinki (1964), and of its amendments, adopted by the 29th, 35th, 41st, 48th, and 52nd General Assemblies of the WMA (Tokyo 1975, Venice 1983, Hong Kong 1989, Somerset West 1996, and Edinburgh 2000), as well as the Clarification Note concerning Paragraph 29 added by the General Assembly of the WMA in Washington 2002, and the Clarification Note concerning Paragraph 30 added by the General Assembly of the WMA in Tokyo 2004.

10.4.2. Other legal considerations

The other legal underpinnings of this clinical trial are the following:

• Topic E6 of the ICH, Guides for Good Clinical Practice, including the subsequent erratum on pass 4, September 1997.

• Directive 2001/20/CE (April 4, 2001).

• Directive of the European Commission 2005/28/EC (April 8, 2005).

• National regulatory requirements / guides for countries participating in clinical trials.

The medical expert and all of the researchers will be provided by the investigator with an up-to-date brochure fully detailing the state of both preclinical and clinical knowledge regarding the medication to be used in the study. Whenever new information is available, an updated version of the brochure, or an addendum to it, will be added to investigators’ file.

10.4.3. Approval of regulatory authorities

No patients may be included in the study until all of the requirements of the national health authorities have been met. The trial will commence only following approval by the relevant ethical committee and the agreement of the corresponding national health authority.

**11. ACCOUNTABILITY**

The sponsor, the researchers, and all of the personnel involved in the clinical trial must be in agreement to carry it out under the Good Clinical Practice of the ICH.

In accordance with Spanish legislation (R.D. 223/2004) and the directives of the European Community (91/507/CEE), all clinical trials, and in all their phases, including those examining bioavailability and bioequivalence, are to be carried out in line with the norms of Good Clinical Practice (GCP).

These norms are designed to help insure that clinical investigation is carried out in line with the highest quality standards, following ethical criteria based on the Helsinki Declaration and subsequent development, and with a guaranteed medical team. The GCP guidelines cover the design, management, performance, compliance, monitoring, auditing, recording of data, analysis, and handling of information in clinical trials to insure that the data and results obtained are correct and credible, and to protect the rights, integrity, and confidentiality of trial subjects.

The ICH (International Conference on Harmonisation of Technical Requirements for Registration of Pharmaceuticals for Human Use) Good Clinical Practice principles are as follows:

1. Clinical trials should be conducted in accordance with the ethical principles that have

their origin in the Declaration of Helsinki, and that are consistent with GCP and the

applicable regulatory requirement(s).

2. Before a trial is initiated, foreseeable risks and inconveniences should be weighed against

the anticipated benefit for the individual trial subject and society. A trial should be

initiated and continued only if the anticipated benefits justify the risks.

3. The rights, safety, and well-being of the trial subjects are the most important

considerations and should prevail over interests of science and society.

4. The available nonclinical and clinical information on an investigational product should be

adequate to support the proposed clinical trial.

5. Clinical trials should be scientifically sound, and described in a clear, detailed protocol.

6. A trial should be conducted in compliance with the protocol that has received prior

institutional review board (IRB)/independent ethics committee (IEC) approval/favorable

opinion.

7. The medical care given to, and medical decisions made on behalf of, subjects should

always be the responsibility of a qualified physician or, when appropriate, of a qualified

dentist.

8. Each individual involved in conducting a trial should be qualified by education, training,

and experience to perform his or her respective task(s).

9. Freely given informed consent should be obtained from every subject prior to clinical trial

participation.

10. All clinical trial information should be recorded, handled, and stored in a way that allows

its accurate reporting, interpretation and verification.

11. The confidentiality of records that could identify subjects should be protected, respecting

the privacy and confidentiality rules in accordance with the applicable regulatory

requirement(s).

12. Investigational products should be manufactured, handled, and stored in accordance with

applicable good manufacturing practice (GMP). They should be used in accordance with

the approved protocol.

**11.1. Direct access to the source documents in accordance with the ICH GCP guidelines**

In accordance with the ICH GCP guidelines, researchers and research centers must provide direct access to source data and documents to allow for activity related to trial monitoring, auditing, independent ethical committee (IEC) review, and legally mandated inspection. All participants have consented (by signing their written informed consent) to direct access to their original medical records for trial-related monitoring, IEC review, and inspection. The contents of the protocol must include identification of all the data to be recorded directly in the CRF (that is, without prior written or electronic recording) which are to be considered source information.

The patient file must include the following information as a minimum:

• Patient demographic information (name, address, date of birth, sex).

• Height and weight (baseline).

• Underlying study illness, date of initial symptoms, and date of first diagnosis of study illness.

• Results of prior endoscopic and histological examinations, if appropriate.

• Medications taken for the illness in question.

• Concomitant illnesses and medications taken for them.

• Proof that the patient is taking part in the study.

• Date of each visit.

• Results of explorations carried out during this trial.

• Activity of the study illness / general state of the patient at each visit.

• All laboratory findings generated in this study.

• Adverse events and adverse drug reactions.

• Reasons for withdrawal, if appropriate.

All other variables may be entered directly into the CRF and will be considered source data.

**11.2. Filing of essential documents**

11.2.1. Investigator

The investigator must keep his or her copies of the CRFs, the signed informed consent forms, the identification list of patients, and the patient records with the original data, for a minimum period of 15 years.

11.2.2. Sponsor

The sponsor will keep all the CRFs and all additional documentation related to the trial for a minimum period of 15 years.

**12. ADDITIONAL FACTORS**

**12.1. PROCEDURES FOR AMENDING THE PROTOCOL**

Any modification of the present protocol that does not involve a substantial change may be made by common consensus, in writing, between the principal investigator and the sponsor.

In the event that the modification(s) involve substantial changes in the original protocol, they must be submitted to the IEC for consideration.

**12.3. TRIAL CANCELLATION**

In the event of cancellation of the trial, the IEC of the hospital must be informed of the reasons for the cancellation.

**13. STATISTICAL ANALYSIS**

The calculation for the sample size is based on the main variable of efficacy—that is, the rate of recidivism at 6 months. On the basis of results of previous studies it was determined that the rate of recidivism with the reintroduction of gluten would be 80%, and with placebo 20%. Assuming a 10% patient drop-out rate, an alpha value of 5%, and statistical power (beta) of 80%, for a bilateral test it was calculated that 10 patients would be needed for each patient group (bearing in mind that analyzing the results would require a Chi-square test with Yates correction). The total number, then, is 20 patients.

Excluded from this analysis are those subjects presenting significant departures from the protocol.

All of the evaluations of the primary and secondary variables, both of efficacy and of safety, are understood in the exploratory sense (alpha=0.05, bilateral).

The Chi-square test with Yates correction will be used for independent groups and the Fisher exact test for qualitative variables. For intergroup comparison of the quantitative variables, the Student-t test will be used for parametric variables, and the Mann-Whitney U-test for non-parametric variables.

The finding of values for differences of p less than 0.05 will be considered significant. The rates for clinical remission between treatment groups will be expressed proportionally with the corresponding bilateral interval of confidence of 95%, as an estimation of therapeutic effect.

**14. REFERENCES**

1. Fine K, Schiller LR. AGA technical review of the evaluation and management of chronic diarrhea. Gastroenterology 1999; 116:1464-86.
2. Thomas PD, Forbes A, Green J, Howdle P, Long R, Playford R, Sheridan M, Stevens R, Valori R, Walters J, Addison GM, Hill P, Brydon G. Guidelines for the investigation of chronic diarrhoea, 2^nd^ edition. Gut 2003; 52 (suppl V):v1-v15.
3. Thompson WG, Longstreth G, Drossman DA, Heaton K, Irvine EJ, Muller-Lissner S. Functional bowel disorders and functional abdominal pain. In: Drossman A, Corazziari E, Talley NJ, Thompson WG, Whitehead WE, eds. Rome II: The functional gastrointestinal disorders. Degnon Associates, McLean, VA, USA; 2000:351-432.
4. Mein SM, Ladabaum U. Serological testing for coeliac disease in patients with symptoms of irritable bowel syndrome: a cost-effectiveness analysis. Aliment Pharmacol Ther. 2004 Jun 1;19(11):1199-210.
5. Spiegel BM, DeRosa VP, Gralnek IM, Wang V, Dulai GS. Testing for celiac sprue in irritable bowel syndrome with predominant diarrhea: a cost-effectiveness analysis. Gastroenterology 2004; 126:1721-36.
6. Wahnschaffe U, Ullrich R, Riecken EO, Schulzke JD. Celiac disease-like abnormalities in a subgroup of patients with irritable bowel syndrome. Gastroenterology 2001; 121:1329-38.
7. Ciclitira PJ. AGA technical review on celiac sprue. Gastroenterology 2001; 120:1526-1540.
8. Wahab PJ, Meijer JWR, Goerres MS, Mulder CJ. Coeliac disease: Changing views on gluten-sensitive enteropathy. Scand J Gastroenterol 2002; 236 (37 Suppl):60-5.
9. Tursi A, Brandimarte G. The symptomatic and histologic response to a gluten-free diet in patients with borderline enteropathy. J Clin Gastroenterol 2003; 36:13-7.
10. Picarelli A, Maiuri L, Mazzilli C, Coletta S, Ferrante P, Di Giovambattista F, Greco M, Torsoli A, Auricchio S. Gluten-sensitive disease with mild enteropathy. Gastroenterology 1996; 111:608-16.
11. Cooper BTT, Holmes GKT, Ferguson R, Thompson RA, Allan RN, Cooke WT. Gluten-sensitive diarrhea without evidence of celiac disease. Gastroenterology 1980; 79:801-6.
12. Rostami K, Kerckhaert J, Tiemessen R von Blomberg BM, Meijer JW, Mulder CJ. Sensitivity of antiendomysium and antigliadin antibodies in untreated celiac disease: disappointing in clinical practice. Am J Gastroenterol 1999; 94:888-94.
13. Tursi A, Brandimarte G, Giorgetti G, Gigliobianco A, Lombardi D, Gasbarrini G. Low prevalence of antigliadin and antiendomysium antibodies in subclinical/silent celiac disease. Am J Gastroenterol 2001;96:1507-10.
14. Dickey W, Hughes DF, McMillan SA. Reliance on serum endomysial antibody testing underestimates the true prevalence of coeliac disease by one fifth. Scand J Gastroenterol 2000; 95:712-4.
15. Esteve M, Rosinach M, Fernández-Bañares F, Farré C, Salas A, Alsina M, Vilar P, Abad-Lacruz A, Forné M, Mariné M, Santaolalla R, Espinós JC, Viver JM. Spectrum of gluten sensitive enteropathy in first degree relatives of celiac patients: clinical relevance of lymphocytic enteritis. Gut 2006; 55:1739-45.
16. Karell K, Louka AS, Moodie SJ, et al. HLA types in celiac disease patients not carrying the DQA1*05-DQB1*02 (DQ2) heterodimer: Results from the European genetics cluster on celiac disease. Human Immunol 2003; 64:469-77.
17. Fernández-Bañares F, Esteve M, Salas A, Alsina M, Farré C, González C, Buxeda M, Forné M, Rosinach M, Espinós JC, Viver JM. Systematic evaluation of the causes of chronic watery diarrhea with functional characteristics. Am J Gastroenterol 2007; 102:2520-8.

**APPENDIX 1**

**Gluten 10 g single-dose product characteristics**

**Xalabarder Pharmacy (Pujades 247, Barcelona)**

1. **PRODUCT DESCRIPTION**

Gluten 10g single-dose.

1. **COMPOSITION**

This product consists of 10g of gluten per unit.

1. **PRESENTATION**

Single-dose sachets with 10g of product.

Boxes of 60 sachets.

6 boxes for each treatment.

1. **RAW MATERIAL**

Raw material purchased from Fagron Ibèrica.

The certificate of analysis lists the following assays and results:

|  | **GLUTEN** | |
| --- | --- | --- |
| **ASSAYS** | | **RESULTS** |
| **Loss through desiccation** | | 7.8% |
| **pH** | | <5 |
| **Total ash** | | 0.9% |
| **Proteins** | | 82 |
| **Fiber** | | 0,5 |
| **Fatty material** | | 1.3 |
| **Carbohydrates** | | 9 |
|  | | **SPECIFICATION COMPLEX** |

1. **PACKAGED MATERIAL**

Thermally sealed aluminum and cellulose Surling-type blister pack.

Sachets are marked with production reference number and expiration date. As per request they do not indicate composition of contents.

Boxes carry the following label:

1. **EXPIRATION**

Product expiration date: 29/5/2012

1. **PREPARATION**

Date: 29/5/2009

Reference: 820055963

Approval: Prepared using the standards for preparation of powders for single-dose oral use. Passed quality control and marked as ‘approved’.

1. **INDICATION**

The product was requested by the Mútua de Terrassa Pharmacy Service for clinical trial EL-DSG/01/01: gluten/maltodextrin.

1. **SIGNATURE**

| **Technical Director** | **Quality Guarantee Unit** |
| --- | --- |
| Eduard Xalabarder | Roser Masanas |

**APPENDIX 2**

**Maltodextrin 10 g single-dose product characteristics**

**Xalabarder Pharmacy (Pujades 247, Barcelona)**

1. **PRODUCT DESCRIPTION**

Maltodextrin 10g single-dose.

1. **COMPOSITION**

This product consists of 10g of maltodextrin per unit.

1. **PRESENTATION**

Single-dose sachets with 10g of product.

Boxes of 60 sachets.

6 boxes for each treatment.

1. **RAW MATERIAL**

Raw material purchased from Fagron Ibèrica.

The certificate of analysis lists the following assays and results:

|  | **MALTODEXTRIN** | |
| --- | --- | --- |
| **ASSAYS** | | RESULTS |
| **Identification** | | Passed |
| **Loss through desiccation** | | 4.74% |
| **Heavy metals** | | <10ppm |
| **Sulphur ash** | | <0.5% |
| **pH** | | 6.02 |
| **Sulphur dioxide** | | <5ppm |
| **Total airborne material** | | <10ufc/g |
| **Fungi and yeast** | | <10ufc/g |
| **E. Coli** | | Passed |
| **Salmonella** | | Passed |
| **Dextrose** | | Passed |
|  | | **SPECIFICATION COMPLEX** |

1. **PACKAGED MATERIAL**

Thermally sealed aluminum and cellulose Surling-type blister pack.

Sachets are marked with production reference number and expiration date. As per request they do not indicate composition of contents.

Boxes carry the following label:

1. **EXPIRATION**

Product expiration date: 29/5/2012

1. **PREPARATION**

Date: 29/5/2009

Reference: 820055964

Approval: Prepared using the standards for preparation of powders for single-dose oral use. Passed quality control and marked as ‘approved’.

1. **INDICATION**

The product was requested by the Mútua de Terrassa Pharmacy Service for clinical trial EL-DSG/01/01: gluten/maltodextrin.

1. **SIGNATURE**

| **Technical Director** | **Quality Guarantee Unit** |
| --- | --- |
| Eduard Xalabarder | Roser Masanas |

**APPENDIX 3**

**Patient quality of life**

Patients will answer a total of 36 questions (elements) concerning the effect of the illness on their quality of life (QOL). Each element is scored on a scale of 5 points, from 1 (poor QOL) to 5 (good QOL). The overall possible score on this GIQLI will therefore range from 0 to 180.

In order to facilitate interpretation, the total for the responses for each scale (symptoms, physical well-being, emotional well-being, and social relations), divided by the number of questions on each scale, will yield the score for the scale. The overall GIQLI score is the sum of the scores for all the scales.

The questions and their pertinence to each of the four scales are as follows:

**The Gastrointestinal Quality of Life Index (GIQLI)**

**1. How often during the past 2 weeks have you had pain in the abdomen?**

1. *All of the time* **2.** *Most of the time* **3.** *Some of the time* **4.** *Almost never* **5.** *Never*

*(Symptoms)*

**2. How often during the past 2 weeks have you had a feeling of fullness in the upper abdomen?**

*All of the time* ***2****. Most of the time* ***3****. Some of the time* ***4****. Almost never* ***5****. Never*

*(Symptoms)*

**3. How often during the past 2 weeks have you had bloating (sensation of too much gas in the abdomen)?**

***1.*** *All of the time* ***2****. Most of the time* ***3****. Some of the time* ***4****. Almost never* ***5****. Never*

*(Symptoms)*

**4. How often during the past 2 weeks have you been troubled by excessive passage of gas?**

***1.*** *All of the time* ***2****. Most of the time* ***3****. Some of the time* ***4****. Almost never* ***5****. Never*

*(Symptoms)*

**5. How often during the past 2 weeks have you been troubled by strong burping or belching?**

***1.*** *All of the time* **2.** *Most of the time* **3.** *Some of the time* **4.** *Almost never* **5.** *Never*

*(Symptoms)*

**6. How often during the past 2 weeks have you been troubled by gurgling noises from the abdomen?**

***1.*** *All of the time* ***2****. Most of the time* ***3****. Some of the time* ***4****. Almost never* ***5****. Never*

*(Symptoms)*

**7. How often during the past 2 weeks have you been troubled by frequent bowel movements?**

***1.*** *All of the time* **2.** *Most of the time* **3.** *Some of the time* **4.** *Almost never* **5.** *Never*

*(Symptoms)*

**8. How often during the past 2 weeks have you found eating to be a pleasure?**

***1.*** *All of the time* ***2****. Most of the time* ***3****. Some of the time* ***4****. Almost never* ***5****. Never*

*(Symptoms)*

**9. Because of your illness, to what extent have you restricted the kinds of food you eat in the past 2 weeks?**

***1.*** *Very much* ***2****. Much* ***3****. Somewhat* ***4****. A little* ***5****. Not at all*

*(Symptoms)*

**10. During the past 2 weeks, how well have you been able to cope with everyday stresses?**

***1.*** *Very poorly* ***2****. Poorly* ***3****. Moderately* ***4****. Well* ***5****. Very well*

*(Emotional)*

**11. How often during the past 2 weeks have you felt sad or depressed?**

***1.*** *All of the time* **2.** *Most of the time* **3.** *Some of the time* **4.** *Almost never* **5.** *Never*

*(Emotional)*

**12. How often during the past 2 weeks have you been nervous or anxious about your illness?**

***1****. All of the time* **2.** *Most of the time* **3.** *Some of the time* **4.** *Almost never* **5.** *Never*

*(Emotional)*

**13. How often during the past 2 weeks have you been happy with life in general?**

***1.*** *All of the time* **2.** *Most of the time* **3.** *Some of the time* **4.** *Almost never* **5.** *Never*

*(Emotional)*

**14. How often during the past 2 weeks have you felt frustrated?**

***1****. All of the time* **2.** *Most of the time* **3.** *Some of the time* **4.** *Almost never* **5.** *Never*

*(Emotional)*

**15. During the past 2 weeks have you been tired or fatigued?**

***1.*** *All of the time* **2.** *Most of the time* **3.** *Some of the time* **4.** *Almost never* **5.** *Never*

*(Physical)*

**16. During the past 2 weeks have you felt unwell?**

***1.*** *All of the time* **2.** *Most of the time* **3.** *Some of the time* **4.** *Almost never* **5.** *Never*

*(Physical)*

**17. Over the past week, have you woken up in the night?**

1. *Every night* **2.** *5 or 6 nights* **3.** *3 or 4 nights* **4.** *1 or 2 nights* **5.** *Never*

*(Physical)*

**18. Since becoming ill, have you been troubled by changes in your appearance?**

1. *A great deal* **2.** *Moderately* **3.** *Somewhat* **4.** *A little bit* **5.** *Not at all*

*(Physical)*

**19. Because of your illness, how much physical strength have you lost?**

***1.*** *A great deal* **2.** *Moderately* **3.** *Somewhat* **4.** *A little bit* **5.** *Not at all*

*(Physical)*

**20. Because of your illness, to what extent have you lost your endurance?**

***1.*** *A great deal* **2.** *Moderately* **3.** *Somewhat* **4.** *A Little bit* **5.** *Not at all*

*(Physical)*

**21. Because of your illness, to what extent do you feel less fit?**

***1****. A great deal* **2.** *Moderately* **3.** *Somewhat* **4.** *A little bit* **5.** *Not at all*

*(Physical)*

**22. During the past 2 weeks, how much have you been troubled by the medical treatment of your illness?**

***1.*** *Very much* **2.** *Much* **3.** *Somewhat* **4.** *A little* **5.** *Not at all*

*(Medical treatment)*

**23. During the past 2 weeks, how often have you been able to complete your normal daily activities (school, work, household)?**

***1.*** *All of the time* **2.** *Most of the time* **3.** *Some of the time* **4.** *Almost never* **5.** *Never*

*(Social)*

**24. During the past 2 weeks, how often have you been able to take part in your usual patterns of leisure or recreational activities?**

***1.*** *All of the time* **2.** *Most of the time* **3.** *Some of the time* **4.** *Almost never* **5.** *Never*

*(Social)*

**25. To what extent have your personal relations with people close to you (family or friends) worsened because of your illness?**

1. *Very much* **2.** *Much* **3.** *Somewhat* **4.** *A little* **5.** *Not at all*

*(Social)*

**26. To what extent has your sexual life been impaired because of your illness?**

1. *Very much* **2.** *Much* **3.** *Somewhat* **4.** *A little* **5.** *Not at all*

*(Social)*

**27. How often during the past 2 week, have you been troubled by fluid or food coming up into your mouth (regurgitation)?**

***1.*** *All of the time* **2.** *Most of the time* **3.** *Some of the time* **4.** *Almost never* **5.** *Never*

*(Symptoms)*

**28. How often during the past 2 weeks have you felt uncomfortable because of your slow speed of eating?**

***1****. All of the time* **2.** *Most of the time* **3.** *Some of the time* **4.** *Almost never* **5.** *Never*

*(Symptoms)*

**29. During the past 2 weeks have you had trouble swallowing your food?**

***1.*** *All of the time* **2.** *Most of the time* **3.** *Some of the time* **4.** *Almost never* **5.** *Never*

*(Symptoms)*

**30. During the past 2 weeks have you been troubled by urgent bowel movements?**

***1****. All of the time* **2.** *Most of the time* **3.** *Some of the time* **4.** *Almost never* **5.** *Never*

*(Symptoms)*

**31. During the past 2 weeks have you been troubled by diarrhea?**

***1.*** *All of the time* **2.** *Most of the time* **3.** *Some of the time* **4.** *Almost never* **5.** *Never*

*(Symptoms)*

**32. During the past 2 weeks have you been troubled by constipation?**

***1.*** *All of the time* **2.** *Most of the time* **3.** *Some of the time* **4.** *Almost never* **5.** *Never*

*(Symptoms)*

**33. During the past 2 weeks have you been troubled by nausea?**

***1.*** *All of the time* **2.** *Most of the time* **3.** *Some of the time* **4.** *Almost never* **5.** *Never*

*(Symptoms)*

**34. During the past 2 weeks have you been troubled by blood in the stool?**

***1.*** *All of the time* **2.** *Most of the time* **3.** *Some of the time* **4.** *Almost never* **5.** *Never*

*(Symptoms)*

**35. During the past 2 weeks have you been troubled by heartburn?**

***1****. All of the time* **2.** *Most of the time* **3.** *Some of the time* **4.** *Almost never* **5.** *Never*

*(Symptoms)*

**36. During the past 2 weeks have you had trouble controlling bowel movements?**

***1.*** *All of the time* **2.** *Most of the time* **3.** *Some of the time* **4.** *Almost never* **5.** *Never*

*(Symptoms)*

**APPENDIX 4**

**Three-day diet log**

**Instructions**

- You will use this questionnaire to record everything that you eat and drink over a three-day period, including holidays.
- It is very important that you don’t change your normal eating habits.
- To make sure that you don’t forget a meal, it is best to write down what you have consumed right after eating or drinking. Don’t forget to include all the ingredients used in each dish.
- You should also include all meals that you have away from home.
- The questionnaire has two sheets for each day. On the first you should record the dishes that you have and how they were cooked, and then on the second the ingredients and their quantities (in weight or in household measurements: tablespoon, teaspoon, cup, bowl, etc.). Try to estimate the amount of oil in tablespoons or teaspoons.
- Indicate whether the weight of foods is before or after cooking, and with or without inedible parts.
- On each sheet indicate the date and the day of the week.
- At the bottom of the page record the recipes for more complex dishes.
- Don’t forget to include sugar, bread, oil, snacks, soft drinks, alcoholic beverages, sweets, chocolate, nuts, potato chips, and so on.
- When describing food, make sure to indicate the quality and type. For milk, indicate whether it is whole, semi-skimmed, or skimmed. For meat and fish, which variety. For bread, whether it is white or whole. And also include butter and margarine where appropriate.
- If you know the brand name, include it.
- If it is a commercial product, note whether it says ‘gluten-free’ on the packaging.
- Note the type and brand of oil used, if possible.
- Indicate whether the dish was pre-cooked or prepared before eating.
- Finally, make a note of any questions or uncertainties that may arise.

**MEALS DAY ONE**

Name: Code:

Date: Age: Sex:

Day of the week: Hospital (initials):

| Time:  Place: | BREAKFAST |
| --- | --- |
| Time:  Place: | MORNING SNACK |
| Time:  Place: | LUNCH |
| Time:  Place: | AFTERNOON SNACK |
| Time:  Place: | DINNER |
| Time:  Place: | OTHER |

**DAY ONE: INGREDIENTS AND QUANTITIES**

| **FOOD ITEM (BRAND, IF KNOWN)** | **QUANTITY** | **LABELLED ‘GLUTEN-FREE’?** |
| --- | --- | --- |
|  |  |  |
|  |  |  |
|  |  |  |
|  |  |  |
|  |  |  |
|  |  |  |
|  |  |  |
|  |  |  |
|  |  |  |
|  |  |  |
|  |  |  |
|  |  |  |
|  |  |  |
|  |  |  |
|  |  |  |
|  |  |  |
|  |  |  |
|  |  |  |
|  |  |  |
|  |  |  |
|  |  |  |
|  |  |  |
|  |  |  |
|  |  |  |
|  |  |  |
|  |  |  |
|  |  |  |
|  |  |  |
|  |  |  |
|  |  |  |
|  |  |  |
|  |  |  |
|  |  |  |
|  |  |  |
|  |  |  |
|  |  |  |
|  |  |  |
|  |  |  |
|  |  |  |
|  |  |  |
|  |  |  |
|  |  |  |
| **FOOD ITEM (BRAND, IF KNOWN)** | **QUANTITY** | **LABELLED ‘GLUTEN-FREE’?** |
|  |  |  |
|  |  |  |
|  |  |  |
|  |  |  |
|  |  |  |
|  |  |  |
|  |  |  |
|  |  |  |
|  |  |  |
|  |  |  |
|  |  |  |
|  |  |  |
|  |  |  |
|  |  |  |
|  |  |  |
|  |  |  |
|  |  |  |
|  |  |  |
|  |  |  |
|  |  |  |
|  |  |  |
|  |  |  |
|  |  |  |
|  |  |  |
|  |  |  |
|  |  |  |
|  |  |  |
|  |  |  |
|  |  |  |
|  |  |  |
|  |  |  |
|  |  |  |
|  |  |  |
|  |  |  |
|  |  |  |
|  |  |  |
|  |  |  |
|  |  |  |
|  |  |  |
|  |  |  |
|  |  |  |
|  |  |  |
|  |  |  |
|  |  |  |

**MEALS DAY TWO**

Date:

Day of the week:

| Time:  Place: | BREAKFAST |
| --- | --- |
| Time:  Place: | MORNING SNACK |
| Time:  Place: | LUNCH |
| Time:  Place: | AFTERNOON SNACK |
| Time:  Place: | DINNER |
| Time:  Place: | OTHER |

**DAY TWO: INGREDIENTS AND QUANTITIES**

| **FOOD ITEM (BRAND, IF KNOWN)** | **QUANTITY** | **LABELLED ‘GLUTEN-FREE’?** |
| --- | --- | --- |
|  |  |  |
|  |  |  |
|  |  |  |
|  |  |  |
|  |  |  |
|  |  |  |
|  |  |  |
|  |  |  |
|  |  |  |
|  |  |  |
|  |  |  |
|  |  |  |
|  |  |  |
|  |  |  |
|  |  |  |
|  |  |  |
|  |  |  |
|  |  |  |
|  |  |  |
|  |  |  |
|  |  |  |
|  |  |  |
|  |  |  |
|  |  |  |
|  |  |  |
|  |  |  |
|  |  |  |
|  |  |  |
|  |  |  |
|  |  |  |
|  |  |  |
|  |  |  |
|  |  |  |
|  |  |  |
|  |  |  |
|  |  |  |
|  |  |  |
|  |  |  |
|  |  |  |
|  |  |  |
|  |  |  |
|  |  |  |
| **FOOD ITEM (BRAND, IF KNOWN)** | **QUANTITY** | **LABELLED ‘GLUTEN-FREE’?** |
|  |  |  |
|  |  |  |
|  |  |  |
|  |  |  |
|  |  |  |
|  |  |  |
|  |  |  |
|  |  |  |
|  |  |  |
|  |  |  |
|  |  |  |
|  |  |  |
|  |  |  |
|  |  |  |
|  |  |  |
|  |  |  |
|  |  |  |
|  |  |  |
|  |  |  |
|  |  |  |
|  |  |  |
|  |  |  |
|  |  |  |
|  |  |  |
|  |  |  |
|  |  |  |
|  |  |  |
|  |  |  |
|  |  |  |
|  |  |  |
|  |  |  |
|  |  |  |
|  |  |  |
|  |  |  |
|  |  |  |
|  |  |  |
|  |  |  |
|  |  |  |
|  |  |  |
|  |  |  |
|  |  |  |
|  |  |  |
|  |  |  |

**MEALS DAY THREE**

Date:

Day of the week:

| Time:  Place: | BREAKFAST |
| --- | --- |
| Time:  Place: | MORNING SNACK |
| Time:  Place: | LUNCH |
| Time:  Place: | AFTERNOON SNACK |
| Time:  Place: | DINNER |
| Time:  Place: | OTHER |

**DAY THREE: INGREDIENTS AND QUANTITIES**

| **FOOD ITEM (BRAND, IF KNOWN)** | **QUANTITY** | **LABELLED ‘GLUTEN-FREE’?** |
| --- | --- | --- |
|  |  |  |
|  |  |  |
|  |  |  |
|  |  |  |
|  |  |  |
|  |  |  |
|  |  |  |
|  |  |  |
|  |  |  |
|  |  |  |
|  |  |  |
|  |  |  |
|  |  |  |
|  |  |  |
|  |  |  |
|  |  |  |
|  |  |  |
|  |  |  |
|  |  |  |
|  |  |  |
|  |  |  |
|  |  |  |
|  |  |  |
|  |  |  |
|  |  |  |
|  |  |  |
|  |  |  |
|  |  |  |
|  |  |  |
|  |  |  |
|  |  |  |
|  |  |  |
|  |  |  |
|  |  |  |
|  |  |  |
|  |  |  |
|  |  |  |
|  |  |  |
|  |  |  |
|  |  |  |
|  |  |  |
|  |  |  |
| **FOOD ITEM (BRAND, IF KNOWN)** | **QUANTITY** | **LABELLED ‘GLUTEN-FREE’?** |
|  |  |  |
|  |  |  |
|  |  |  |
|  |  |  |
|  |  |  |
|  |  |  |
|  |  |  |
|  |  |  |
|  |  |  |
|  |  |  |
|  |  |  |
|  |  |  |
|  |  |  |
|  |  |  |
|  |  |  |
|  |  |  |
|  |  |  |
|  |  |  |
|  |  |  |
|  |  |  |
|  |  |  |
|  |  |  |
|  |  |  |
|  |  |  |
|  |  |  |
|  |  |  |
|  |  |  |
|  |  |  |
|  |  |  |
|  |  |  |
|  |  |  |
|  |  |  |
|  |  |  |
|  |  |  |
|  |  |  |
|  |  |  |
|  |  |  |
|  |  |  |
|  |  |  |
|  |  |  |
|  |  |  |
|  |  |  |
|  |  |  |

**APPENDIX 5.**

**Gluten-free diet compliance assessment**

**Made by a dietician expert in coeliac disease**

|  |  | **Always**  **(0)** | **Almost always**  **(1)** | **Sometimes**  **(2)** | **Rarely**  **(3)** | **Never**  **(4)** |
| --- | --- | --- | --- | --- | --- | --- |
| **1** | Eats at restaurants that cater to coeliac sufferers or asks appropriate questions when eating away from home |  |  |  |  |  |
| **2** | Observes the policy of ‘If in doubt, don’t eat it.’ |  |  |  |  |  |
| **3** | Examines medicines, supplements, and other healthcare products that are taken through the nose, the eyes, or the mouth |  |  |  |  |  |
| **4** | Reads and understands the labels on products |  |  |  |  |  |
| **5** | Eliminates the sources of possible contamination from the table and the kitchen |  |  |  |  |  |
|  |  | **Always**  **(4)** | **Almost**  **(3)** | **Sometimes**  **(2)** | **Rarely**  **(1)** | **Never**  **(0)** |
| **6** | Eats oats (contaminated) |  |  |  |  |  |
| **7** | Knowingly eats or does not eat gluten (according to the dietary questionnaire and lifestyle) |  |  |  |  |  |
| **8** | Trusts partner or family member to correctly carry out gluten-free diet |  |  |  |  |  |

|  | **Social questions** | **Yes** | **No** |
| --- | --- | --- | --- |
| **1** | Shows poor adherence/motivation due to denial, anxiety, finances, family or social problems |  |  |
| **2** | Has mental or behavioral problems that impede adherence to the diet |  |  |
| **3** | Has language problems that impede adherence to the diet |  |  |
| **4** | Eats products that contain gluten for religious reasons (communion, Passover, etc.) |  |  |
| **5** | Needs to be constantly reminded of hidden gluten in foods |  |  |

**Excellent**: 0 to 7, and on social question answers ‘no’ to all

**Good**: 8 to 15, and on social questions answers ‘no’ to all

**Average**: 16 to 24, and on social questions answers ‘yes’ to 2, or ‘good’ to 1 and 1 social question ‘yes’

**Poor**: 25 to 32 and social questions: 2 ‘yes’; or ‘average’ and 3 social questions ‘yes’

**Very poor**: Poor and 3 social questions or more ‘yes’

**APPENDIX 6.**

**Patient global symptom assessment**

**How to complete the analogue visual scale:**

- Please read the following questions very carefully.
- For each of the scales below, please mark a single vertical line at the point that best captures your symptoms during the past week.

1. How strong was the feeling of abdominal gas in the past week?

None Very slight Slight Moderate Strong Very strong

2. How strong was the feeling of abdominal bloating in the past week?

None Very slight Slight Moderate Strong Very strong

3. How strong was the feeling of abdominal pain or discomfort in the past week?

None Very slight Slight Moderate Strong Very strong

4. How strong were changes in intestinal regularity (diarrhea) in the past week?

None Very slight Slight Moderate Strong Very strong

**APPENDIX 7**.

Patient information sheet

Lymphocytic enteritis with negative coeliac serology and clinical and histological response to a gluten-free diet: double-blind randomized clinical trial with rechallenge of gluten vs. placebo.

**Study code: EL-DSG/07/2011**

**Dear patient,**

You are invited to take part in a clinical study designed to evaluate the possibility of reintroducing gluten into your diet.

This document is meant to explain the aims and procedures of this study to you. Please read it carefully. If you have any questions, or if there are things that you do not understand, please get in touch with the doctor who is directing the study.

###### PURPOSE AND GENERAL INFORMATION

You have a form of enteropathy that involves sensitivity to gluten, which is why you have been given a gluten-free diet. This condition is seen as a mild form of coeliac disease. You are certainly aware that gluten is a protein contained in certain cereals (wheat, rye, barley, and oats). Intolerance to gluten in your diet produces an illness which in its most common form leads to intestinal villous atrophy, known as coeliac disease. In adults this often involves lesions of the intestine in the form of inflammation of the mucous membrane (lymphocytic enteropathy); this is the kind of gluten sensitivity that you apparently suffer.

However, recent studies have uncovered another kind of intolerance to gluten that is not coeliac, although a reduction of gluten intake does improve the symptoms. This kind of intolerance may not require a strict diet for life; it may be the case that this type of gluten intolerance is reversible. In other words, it might be possible for you to return to a diet that includes gluten without problems. However, today we do not yet have diagnostic tests in clinical practice that can identify which type of gluten intolerance you suffer.

This study aims to investigate whether your gluten intolerance is of a coeliac or non-coeliac nature, and to learn whether the sensitivity to gluten that caused your symptoms remains or has disappeared. To this end we would like to invite you to take part in a dietary study in which we examine what happens with the reintroduction of gluten in comparison to placebo, in 20 patients with this illness. That is, you will be assigned to one of two groups for this dietary study under a double-blind protocol: neither you nor your doctor will know which of the formulas you have been randomly given. You will continue with your gluten-free diet, and every 12 hours you will take a sachet with powder that is either gluten or placebo, to be mixed with your food (yogurt, soup, or puree).

The treatment will last for 6 months. During this time, you will have to see your doctor 5 times. He or she will ask you to carefully keep a diary during the entire period, which you will need to bring to each medical visit. In the diary you will record certain details that are specific to the disease, such as the number of bowel movements and their form (watery, soft, or solid), your pain and abdominal cramps, a record of the regular use of the sachets, and the use of other medication as well.

Both at the beginning and the end of the study you will undergo a blood test and gastroscopy with intestinal biopsy (with sedation if you prefer) to assess the level of intestinal lesion. These samples will also allow us to study immunological characteristics that may help in differentiating between the coeliac and non-coeliac forms of gluten intolerance.

We must inform you that it is possible that your illness may get worse with the reintroduction of gluten, although this will also serve to mark your degree of sensitivity to it. If this happens before the 6 months of the study we will end it early for you (you should expect to participate in the diet study for at least one month’s time). If your condition does not worsen, however, we will expect you to complete the 6 months marked out for the study.

###### POTENTIAL BENEFITS

This study may help us to determine whether your intolerance to gluten is of a coeliac or a non-coeliac nature, and whether, by implication, you will need to follow a strict non-gluten diet for life.

During the study you will undergo periodic reviews; in addition, your medical monitoring will be closer than when using normal therapeutic procedures.

By taking part in this study you will be helping doctors to better understand the immunological analytical parameters of intestinal biopsy which in the future will allow for easier differentiation between the coeliac and non-coeliac forms of gluten intolerance.

###### COSTS OF PARTICIPATING IN THE STUDY

The study sponsor (the digestive service) will absorb the cost of all medical procedures and of the dietary powder that you will be taking. Your participation will not cost you anything.

###### CONFIDENTIALITY

This study is in full compliance with the data protection norms in force in Spain. All data obtained during the study about you will be recorded and scientifically evaluated without disclosing your identity. With the aim of assuring at all times that the study is being carried out in compliance with the legal guidelines, representatives of the local or national healthcare authorities and representatives of the ethical committee of the hospital may examine your medical records and the information gathered in the study. These people will respect the rules of confidentiality. Your personal data will be treated as confidential and will not be made public.

###### VOLUNTARY NATURE OF PARTICIPATION AND WITHDRAWAL FROM THE STUDY

It is of the utmost importance that you understand that your participation in this study is completely voluntary. You are under no obligation to take part in it if you do not wish to do so. You may withdraw from the study at any time without any negative repercussion on your normal medical treatment or your relationship with your doctor and the hospital. If you do decide to withdraw from the study, please contact your physician before doing so.

You should request any further information that you may wish to have before signing the informed consent form.

Your doctor may suspend the dietary intervention at any time if he or she feels that it is in your best interest, or in the interest of best completing the study (for example, if you do not meet the requirements of the study). With your consent, your GP will be informed of your participation in the study. Any decision to terminate the study as a whole before its completion will be at the discretion of the sponsor and the competent authority in this matter.

You may not take part in this study if you have taken part in another study in the 30 days prior to the first visit, if you are planning to participate in another study at the same time, or if you have already participated in the present study.

**Thank you for your collaboration!**

**If you would like further information about this study, would like to clear up any legal questions, or feel that you are in an emergency situation, please contact:**

|  |
| --- |

Physician’s seal (Investigator)

**APPENDIX 8.**

INFORMED CONSENT

Lymphocytic enteritis with negative coeliac serology and clinical and histological response to a gluten-free diet: double-blind randomized clinical trial with rechallenge of gluten vs. placebo.

I, .......................................................................................................

(First name and surnames, printed in capital letters)

- - - have read the consent form that I was given.
    - have been able to ask questions about the study
    - have received ample information about the study
    - have spoken with:

(name of the investigator in capital letters)

........................................................................................................

I fully understand that:

1. my participation is voluntary.
2. this analysis does not offer me any direct benefit.
3. refusing to participate in this study will have no effect on my medical care.
4. the information obtained in this study is confidential.

(Date) (Signature of participant)
