# Supplementary material for: Double-Blind Randomized Clinical Trial: Gluten versus Placebo Rechallenge in Patients with Lymphocytic Enteritis and Suspected Celiac Disease
Source: PLoS One. 2016 Jul 8;11(7):e0157879. doi: 10.1371/journal.pone.0157879 (PMC4938236; doi:10.1371/journal.pone.0157879)
Supplement: S2 Protocol — (DOC) [file pone.0157879.s007.doc]

# PROTOCOLO ENSAYO CLINICO

**Enteropatía linfocítica con serología de celiaquía negativa y respuesta clínica e histológica a la dieta sin gluten: Estudio clínico aleatorizado de reintroducción de gluten vs placebo.**

## Ensayo Fase IV

**Código: EL-DSG/01/01**

### Versión Junio 2011

### HOJA DE CONFORMIDAD

**______________________________________________________________________**

**Título del protocolo:**

Enteropatía linfocítica con serología de celiaquía negativa y respuesta clínica e histológica a la dieta sin gluten: Estudio clínico aleatorizado de reintroducción de gluten vs placebo.

**Promotor:**

Servicio de Aparato Digestivo

Hospital Mutua Terrassa

Plaza Dr Robert, 5

08221 Terrassa (Barcelona)

**Director del proyecto:**

Dr Fernando Fernández Bañares

Médico Adjunto del Servicio de Aparato Digestivo

Hospital Universitari Mutua Terrassa

Fecha

Firma

Fecha del primer borrador: Febrero 2011

Fecha versión definitiva: Junio 2011

# 1. RESUMEN DEL ESTUDIO

| **Título** | Enteropatía linfocítica con serología de celiaquía negativa y respuesta clínica e histológica a la dieta sin gluten: Estudio clínico aleatorizado de reintroducción de gluten vs placebo. | |
| --- | --- | --- |
| **Código del estudio** | EL-DSG | |
| **Objetivos** | Demostrar la hipersensibilidad al gluten en pacientes con enteropatía linfocítica y respuesta clínica e histológica a la dieta sin gluten a pesar de presentar anticuerpos antitransglutaminasa tisular IgA negativos. | |
| **Tratamiento** | Sobres de gluten (10 g) o placebo (maltodextrina: 10 g): 1 sobre cada 12 horas. | |
| **Diseño del estudio** | Estudio prospectivo, aleatorizado, doble ciego, controlado con placebo, grupos en paralelo, de reintroducción de gluten vs placebo, durante 24 semanas, en pacientes que realizan dieta sin gluten.   - Grupo A: Gluten en polvo (10 g cada 12 horas) - Grupo B: Placebo (10 g cada 12 horas)   Determinación de la clínica digestiva, adherencia a la dieta, serología de celiaca, histología, citometría de flujo y depósitos de anticuerpos antitransglutaminasa tisular en mucosa intestinal de forma basal y después de la reintroducción del gluten o placebo (si reaparece la clínica digestiva a las 4 sem o 12 sem, o al completar el estudio a las 24 sem).. | |
| **Población en estudio** | Criterios de inclusión:  1. Firmar el consentimiento informado,  2. Pacientes con edad ≥ 18 años,  3. Diagnóstico histológico confirmado de enteropatía linfocítica (EL),  5. Estudio genético de celiaquía positivo (HLA-DQ2 y/o HLA-DQ8 positivos),  6. Serología de celiaquía negativa,  7. Respuesta clínica e histológica completa a la dieta sin gluten,  8. Clínica inicial digestiva con o sin manifestaciones extraintestinales. | |
|  | Criterios de exclusión:  1. Pacientes que son incapaces de adherirse al calendario de visitas del estudio u otros requisitos del protocolo a juicio del investigador.  2. Participación en un ensayo clínico en los últimos 30 días, participación simultánea en un ensayo o participación previa en el presente estudio.  3. Antecedentes de enteropatía sensible al gluten con atrofia vellositaria o serología positiva.  4. Pacientes con enteropatía linfocítica y respuesta a la dieta sin gluten inicial que en el momento de la inclusión realicen dieta normal. | |
| **Tamaño de la muestra** | 20 | |
| **Duración del tratamiento** | 24 semanas | |
| **Variables principales de eficacia** | Reaparición de clínica digestiva y recidiva histológica | |
| **Variables secundarias de eficacia** | - Clínica digestiva 4 semanas. - Clínica digestiva 12 semanas. - Citometría de flujo para medición del porcentaje de linfocitos CD3+ gamma-delta+ y CD3- en mucosa intestinal. | - Valoración de depósitos de anticuerpos antitransglutaminasa tisular IgA en la mucosa intestinal. - Anticuerpos séricos IgA anti-transglutaminasa tisular y antiendomisio. |

**2.- INDICE**

1. Resumen del estudio 3
2. Indice 5
3. Información general 6
4. Justificación y objetivos 7
5. Tipo de ensayo clínico y diseño del mismo 8
6. Selección de sujetos 9
7. Descripción del tratamiento 10
8. Desarrollo del ensayo y evaluación de la respuesta 11
9. Acontecimientos adversos 13
10. Aspectos éticos 18
11. Responsabilidades 20
12. Consideraciones adicionales 22
13. Análisis estadístico 23
14. Bibliografía 24

Apéndice 1. Características sobres monodosis Gluten 10 g ics 26

Apéndice 2. Características sobres monodosis Maltodextrin 10 g 28

Apéndice 3. Calidad de vida del paciente 30

Apéndice 4. Registro de alimentos de 3 días 33

Apéndice 5. Valoración del cumplimiento con la dieta sin gluten 43

Apéndice 6. Escalas de valoración de los síntomas 44

Apéndice 7. Hoja de información del paciente 45

Apéndice 8. Hoja de consentimiento informado 50

**3.- INFORMACIÓN GENERAL**

**3.1.- CODIGO**

EL-DSG/01/01

**3.2.- TITULO**

Enteropatía linfocítica con serología de celiaquía negativa y respuesta clínica e histológica a la dieta sin gluten: Estudio clínico aleatorizado de reintroducción de gluten vs placebo.

**3.3.- TIPO DE ENSAYO**

Ensayo clínico en Fase IV.

**3.4.- DESCRIPCIÓN DE LA TERAPIA DEL ESTUDIO**

El gluten es una proteína contenida en los cereales tipo trigo, avena, centeno y cebada y está compuesta por gliadina y glutenina. Por tanto, es ampliamente utilizado en alimentación. La ingesta habitual de gluten en la dieta es de 20 gramos al día.

Se administrará en forma de sobres conteniendo 10 gramos de gluten.

**3.5.- DATOS RELATIVOS AL PROMOTOR**

Servicio de Aparato Digestivo

Hospital Universitari Mutua Terrassa

Plaza Dr Robert, 5

08221 Terrassa (Barcelona)

**3.6.- DIRECTOR TÉCNICO RESPONSABLE DE LA ELABORACIÓN DE LAS MUESTRAS**

Jaume Llorente

Farmacia Xalabarder

Terrassa

**3.7.- IDENTIFICACIÓN DEL MONITOR Y UNIDAD DE GARANTIA DE CALIDAD**

##### MONITORES

Maria Esteve Comas

Médico Gastroenterólogo

Servicio de Aparato Digestivo

Hospital Mutua Terrassa

**3.8.- DURACIÓN PREVISTA DEL ENSAYO**

Período máximo de reclutamiento previsto: 30 meses.

Duración máxima del período de tratamiento: 6 meses.

Duración máxima prevista: 36 meses.

**4.- JUSTIFICACION Y OBJETIVOS DEL ESTUDIO**

**4.1.- JUSTIFICACIÓN**

En los últimos años, el concepto de enfermedad celiaca (EC) ha sufrido una profunda revisión. Se ha reconocido un amplio espectro de manifestaciones clínicas y formas de presentación histológica y se ha realizado un importante progreso en la comprensión de los mecanismos genéticos e inmunológicos de la enfermedad. El común denominador para todos los pacientes con EC es la presencia de una combinación variable de manifestaciones clínicas gluten-dependientes, auto-anticuerpos específicos (anti-transglutaminasa tisular 2, antiendomisio), haplotipos HLA-DQ2 y/o DQ8 y diferentes grados de enteropatía desde la infiltración linfocítica del epitelio (enteropatía linfocítica, EL) a la atrofia completa de vellosidades (1).

Por otro lado, existe otra entidad, denominada sensibilidad al gluten no celiaca (SGNC), cuyos límites y posible superposición con la EC están todavía poco definidos (2,3). En la actualidad, la SGNC comprende una serie de alteraciones morfológicas, funcionales e inmunológicas, en ausencia de alguna de las características clave de la EC (enteropatía, haplotipos HLA asociados y presencia de anticuerpos anti-tTG2) pero con respuesta clínica a la exclusión del gluten (4). En este sentido, se han descrito pacientes con SGNC y mucosa sin atrofia de vellosidades, que incluye pacientes sin lesión mucosa, es decir, sin enteropatía (Marsh 0) o pacientes con cambios mínimos en la mucosa, es decir, con enteropatía (EL, Marsh 1). A diferencia de la EC, la SGNC no se asociaría a una predisposición genética, podría ser reversible y la dieta sin gluten probablemente no debería ser tan estricta. En los últimos años, se han diagnosticado en nuestro servicio muchos pacientes de enteropatía sensible al gluten, dentro del espectro de la EC, en base a la presencia de EL, estudio genético de celiaquía positivo y respuesta clínica e histológica a la DSG, a pesar de que la serología específica había sido negativa. En base al conocimiento actual nos planteamos si todos estos pacientes presentan EC, o si un número indeterminado de ellos presentan una SGNC. Valorar la respuesta clínica e histológica a la reintroducción al gluten, así como las determinaciones inmunológicas específicas en la mucosa intestinal, podría permitir realizar un diagnóstico definitivo de EC en estos pacientes. No es fácil realizar una DSG durante toda la vida y, por tanto, el diagnóstico de EC debería de basarse en una evidencia definitiva. Por ello, los resultados del presente estudio serán de gran utilidad para ampliar el espectro de lo que hoy se conoce como EC y tendrán una amplia aplicabilidad en la práctica clínica habitual.

**4.2. - OBJETIVOS**

Demostrar la hipersensibilidad al gluten en pacientes con enteropatía linfocítica y respuesta clínica e histológica a la dieta sin gluten a pesar de presentar anticuerpos antitransglutaminasa tisular IgA negativos.

**5. - TIPO DE ENSAYO CLINICO Y DISEÑO DEL MISMO**

Se trata de un ensayo clínico en fase IV, doble ciego, controlado con placebo de dos vía en paralelo y de asignación aleatoria de los tratamientos.

**5.1. - ALEATORIZACION**

Se realizará una asignación aleatoria de los tratamientos según una proporción 1:1. La asignación se generará mediante ordenador y la medicación se codificará con números consecutivos, para ser dispensada, siguiendo un esquema doble ciego, en la farmacia del hospital a la entrada del sujeto en el estudio.

En el día 1 del estudio cada paciente aleatorizado recibirá un número de participación que se corresponderá con el número de medicación, y que representa un número de aleatorización asociado a un tratamiento específico.

**5.2. - TÉCNICAS DE ENMASCARAMIENTO**

Para el enmascaramiento del suplemento de gluten y el placebo ambos tendrán las mismas características organolépticas. Se administrarán en forma de sobres opacos conteniendo polvo seco. Los envases tendrán el mismo aspecto y estarán codificados con números consecutivos.

**6. - SELECCIÓN DE SUJETOS**

**6.1. - CRITERIOS DE INCLUSIÓN**

1. Firmar el consentimiento informado,

2. Pacientes con edad ≥ 18 años,

3. Diagnóstico histológico confirmado de enteropatía linfocítica (EL),

5. Estudio genético de celiaquía positivo (HLA-DQ2 y/o HLA-DQ8 positivos),

6. Serología de celiaquía negativa,

7. Respuesta clínica e histológica completa a la dieta sin gluten,

8. Clínica inicial digestiva con o sin manifestaciones extraintestinales

*Se define enteritis linfocítica como una cifra de linfocitos intraepiteliales superior a 25 por 100 células epiteliales, utilizando para el recuento técnicas de inmunohistoquímica con anticuerpos monoclonales anti-CD3.

**6.2. - CRITERIOS DE EXCLUSION**

1. Pacientes que son incapaces de adherirse al calendario de visitas del estudio u otros requisitos del protocolo a juicio del investigador.

2. Participación en un ensayo clínico en los últimos 30 días, participación simultánea en un ensayo o participación previa en el presente estudio.

3. Antecedentes de enteropatía sensible al gluten con atrofia vellositaria o serología positiva.

4. Pacientes con enteropatía linfocítica y respuesta a la dieta sin gluten inicial que en el momento de la inclusión realicen dieta normal.

**6.3. - NUMERO DE SUJETOS PREVISTO**

El tamaño calculado de la muestra es de 20 pacientes (10 pacientes en cada grupo de tratamiento) que tienen que ser evaluables en el análisis por intención de tratar.

**7.- DESCRIPCIÓN DEL TRATAMIENTO**

Los sujetos incluidos en el estudio se distribuirán aleatoriamente en dos grupos terapéuticos. El código asignado aleatoriamente adjudicará una de las dos opciones de tratamiento a saber:

**Grupo 1 (Grupo sin gluten)**: Recibirán una dieta sin gluten y suplementos de placebo (10 gramos de maltodextrina en polvo), 1 sobre cada 12 horas, mezclado con los alimentos (purés, sopas).

**Grupo 2 (Grupo con gluten)**: Recibirán una dieta sin gluten y suplementos de gluten (10 gramos de gluten en polvo), 1 sobre cada 12 horas, mezclado con los alimentos (purés, sopas).

**7.1.- TRATAMIENTOS CONCOMITANTES**

Cualquier medicación que deba ser administrada se anotará en la hoja correspondiente del cuaderno de recogida de datos.

**7.2.- MEDIDAS PARA VALORAR EL CUMPLIMIENTO**

La estimación del cumplimiento se realizará mediante el recuento del número de sobres devueltos en cada visita y mediante el registro en el diario del paciente.

Se administrará por escrito una dieta sin gluten estándar. Los pacientes serán visitados por una dietista o experto(a) en nutrición para valorar el cumplimiento estricto de la dieta, con visitas mensuales durante toda la duración del estudio.

**7.3.- NORMAS PARA EL MANEJO DE LA MEDICACIÓN A ESTUDIO**

La distribución de las muestras para el ensayo clínico se realizará a través del Servicio de Farmacia. El Farmacéutico/Investigador deberá completar y devolver al promotor la hoja de entrega de las distintas terapias verificando la recepción de los sobres; se responsabilizará de su correcta conservación y administración, controlando la medicación sobrante al final del estudio. Toda la medicación para el estudio deberá ser almacenada en un lugar seguro mientras dure el mismo.

Toda la medicación será contabilizada. Al final del estudio, una copia del inventario de la medicación deberá ser archivada por el investigador. La medicación sobrante será devuelta por el Servicio de Farmacia correspondiente al promotor, quien la destruirá.

**7.4.- EMPAQUETADO Y ETIQUETADO**

La medicación será entregada en forma de sobres conteniendo gluten o maltodextrina según la aleatorización que corresponda al sujeto. Los sobres irán numerados por orden correlativo y deberán ser asignados en cada centro por riguroso orden según la inclusión de sujetos en el estudio. El evaluador desconocerá en todo momento la clave de aleatorización.

**8.- DESARROLLO DEL ENSAYO Y EVALUACIÓN DE LA RESPUESTA**

**8.1.- VARIABLES PRINCIPAL Y SECUNDARIAS DE EVALUACIÓN**

**Criterios de eficacia**

- Variable principal de valoración de eficacia: Reaparición de clínica digestiva (24 semanas)
- Asimismo, como variables secundarias de valoración de la eficacia se valorarán:
  - Clínica digestiva 4 semanas.
  - Clínica digestiva 12 semanas.
  - Citometría de flujo para medición del porcentaje de linfocitos CD3+ gamma-delta+ y CD3- en mucosa intestinal.
  - Valoración de depósitos de anticuerpos antitransglutaminasa tisular IgA en la mucosa intestinal.
  - Anticuerpos séricos IgA anti-transglutaminasa tisular y antiendomisio.
  - Calidad de vida según el cuestionario GIQLI (Apéndice 3).
  - Cambios histológicos en la biopsia de duodeno distal.

**Criterios de seguridad**

La dieta sin gluten está exenta de efectos secundarios por ella misma. De todas maneras se efectuará un registro de efectos adversos habitual durante la duración del estudio.

**8.2.- DESARROLLO DEL ENSAYO, NUMERO DE VISITAS, EXPLORACIONES COMPLEMENTARIAS**

Los sujetos se seleccionarán según los criterios de inclusión y exclusión establecidos en el protocolo. En el caso de que el paciente cumpla los criterios de inclusión y ninguno de los criterios de exclusión se le informará de la existencia del presente estudio, de sus características, las ventajas e inconvenientes y la conveniencia de su colaboración expresa. Se le entregará una hoja informativa y se solicitará su consentimiento para participar en el mismo.

El paciente que acepte participar en el estudio recibirá un número de código en base al cual le corresponderá de forma aleatoria uno de los dos tipos de tratamiento.

A partir del inicio del mismo, el sujeto será visitado en los meses 1, 3 y 6 del inicio del ensayo de forma estipulada, y más a menudo, si fuera preciso.

En las visitas pre-inclusión se efectuará determinación analítica, serología de celiaquía, estudio genético de celiaquía (HLA-DQ2/HLA-DQ8, si no estuvieran hechos), biopsia de duodeno distal para anatomía patológica y citometría de flujo.

**8.3 – DESCRIPCIÓN DE LOS METODOS EMPLEADOS PARA LA EVALUACIÓN DE LA RESPUESTA**

La respuesta clínica se valorará mediante escalas analógicas visuales de 0 a 100 de forma basal y en cada visita hasta la visita a los 6 meses, para los síntomas dolor abdominal, hinchazón abdominal, diarrea, flatulencia, (puntuación total entre 0 y 400 puntos).

**8.4 - CRITERIOS DE RETIRADA**

Todos los pacientes pueden retirarse del estudio en cualquier momento sin que ello conlleve desventajas personales y sin tener que dar ninguna explicación. Deben documentarse en el CRD el momento de la retirada, los resultados disponibles hasta ese momento y, si se conocen, las causas que motivaron dicha retirada.

El investigador también puede retirar al paciente del estudio tras considerar la relación riesgo/beneficio, si ya no considera justificable la ulterior participación del paciente en el estudio. Deben documentarse en el CRD la fecha y la causa principal de la retirada, así como las observaciones disponibles en el momento de la misma. Entre los motivos que pueden llevar a retirar a un paciente del estudio se encuentran los siguientes (debe determinarse el motivo primario):

• falta de eficacia de la terapia del estudio, p.ej.:

– progresión de la enfermedad del estudio en comparación con la situación basal

(durante las 6 semanas subsiguientes).

– necesidad de administrar una medicación concomitante prohibida para el tratamiento de la enfermedad del estudio.

• acontecimientos adversos intolerables.

• falta de colaboración por parte del paciente, p.ej.:

– solicitud del paciente.

– falta de cumplimiento terapéutico o de asistencia a las visitas intermedias según el programa acordado.

– motivos técnicos / logísticos (p.ej., un cambio en el lugar de residencia).

– embarazo o lactancia existentes o planificadas.

• otros motivos (es preciso anotar el motivo), p.ej.:

- diagnóstico diferente al de la enfermedad del estudio.

En todos los pacientes que finalicen el estudio prematuramente deberá efectuarse una visita en un plazo máximo de 7 días tras la última administración de la medicación del estudio. Esta visita debe realizarse en calidad de visita final y documentarse en el CRD.

El investigador continuará observando a los pacientes retirados del estudio como consecuencia de la aparición de AA intolerables hasta que los hallazgos se hayan esclarecido.

**9.- ACONTECIMIENTOS ADVERSOS**

**9.1.-** **DEFINICIONES**

9.1.1 Acontecimientos adversos

Los acontecimientos adversos (AA) se registraran en cada visita de control en las notas del paciente (documento fuente) y en el CRD.

Un AA es todo incidente medico desfavorable ocurrido a un paciente o participante en una investigación clínica al que se le ha administrado un producto farmacéutico y que no guarda necesariamente una relación causal con este tratamiento. Un AA puede ser, por lo tanto, cualquier signo (incluido un hallazgo de laboratorio anómalo), síntoma o enfermedad desfavorable y accidental asociado en el tiempo con el uso de un medicamento, guarde relación o no con el mismo, como, p.e.:

. cualquier nuevo diagnóstico

. cualquier síntoma que requiera esclarecimiento médico o lleve al ingreso

hospitalario del paciente (cirugía o accidente)

. cualquier presunta reacción medicamentosa adversa

. cualquier síntoma que aparezca en las notas médicas del paciente

. cualquier evento relacionado en el tiempo con la administración de la medicación

del estudio que afecte a la salud del paciente (incluidos cambios en los valores de

laboratorio).

9.1.2 Acontecimientos adversos serios

Un acontecimiento adverso serio (AAS) es todo incidente médico desfavorable que en cualquier dosis:

. provoca la muerte.

. es potencialmente mortal.

. requiere el ingreso hospitalario del paciente o la prolongación de un ingreso

preexistente.

. da lugar a una discapacidad / incapacidad persistente o significativa.

. provoca una anomalía congénita / defecto de nacimiento.

Los acontecimientos adversos no serios son todos los AA que no se amoldan a ninguna de las categorías descritas.

9.1.3 Reacción medicamentosa adversa inesperada

Una reacción medicamentosa adversa (RMA) inesperada es una reacción adversa cuya

naturaleza o gravedad no concuerda con los datos incluidos en la información relativa al producto pertinente.

9.1.3.1 Lista de reacciones medicamentosas adversas esperadas

No se espera ningún acontecimiento adverso por la administración de una dieta sin gluten ni los suplementos de gluten y placebo.

**9.2** **DOCUMENTACIÓN Y NOTIFICACIÓN**

9.2.1 Documentación y notificación de los acontecimientos adversos

Se deberá indicar a los pacientes la necesidad de que contacten con el investigador de forma inmediata en caso de que se produzca algún incidente que pudiera necesitar la aplicación de medidas adecuadas.

Además, en todas las visitas intermedias y en la visita final, el investigador deberá formular una pregunta al respecto en términos generales, sin incidir en ningún síntoma en particular, como, p.e., ¿Ha empeorado su estado de salud desde la última vez que nos vimos? Si la respuesta a esta pregunta es ‘no’, no se realizarán mas preguntas. Si la respuesta es ‘si’, el investigador deberá documentar la naturaleza, el momento, la gravedad, la seriedad y la duración del AA, así como su causalidad.

Para cada AA deben documentarse los siguientes datos:

. Naturaleza del acontecimiento

. Momento de inicio: fecha, hora

. Intervalo entre la última administración de la medicación del estado y el inicio del AA

. Tratamiento concomitante: producto (nombre genérico, indicación, dosis, intervalo

de dosificación, presentación, modo de administración, pauta de administración

. Duración del AA

. Frecuencia

. Gravedad

. Seriedad

. Causalidad

. Medidas

. Evolución

La gravedad del acontecimiento se evalúa de la siguiente manera:

1. Leve: el paciente percibe el acontecimiento/síntoma, pero lo tolera fácilmente y no interfiere con sus actividades cotidianas habituales.
2. Moderado: el acontecimiento/síntoma interfiere con las actividades cotidianas habituales.
3. Grave: el acontecimiento/síntoma impide las actividades cotidianas habituales.

La relación entre un AA y la medicación del estudio se categoriza de acuerdo con la clasificación de la OMS (se deberían satisfacer razonablemente todos los puntos):

. Segura:

Un acontecimiento clínico, incluida una anomalía en una prueba de laboratorio, cuya aparición guarda una relación temporal plausible con respecto a la administración del fármaco y que no puede explicarse por la presencia de una enfermedad concurrente ni por el uso de otros fármacos o sustancias químicas. La respuesta a la retirada del fármaco (eliminación de la exposición) debería tener resultados clínicamente plausibles. El acontecimiento debe ser definitivo farmacológica o fenomenológicamente, para lo que se utilizara un procedimiento de re-exposición satisfactorio, si es necesario.

. Probable / presumible

Un acontecimiento clínico, incluida una anomalía en una prueba de laboratorio, cuya aparición guarda una secuencia temporal razonable con respecto a la administración del fármaco, improbablemente atribuible a una enfermedad concurrente o al uso de otros fármacos o sustancias químicas y que presenta una respuesta clínica razonable al retirar el fármaco (eliminación de la exposición). No se precisa información relativa a la reexposición al fármaco para cumplir esta definición.

. Posible

Un acontecimiento clínico, incluida una anomalía en una prueba de laboratorio, cuya aparición guarda una secuencia temporal razonable con respecto a la administración del fármaco, pero que también podría explicarse por la presencia de una enfermedad concurrente o por el uso de otros fármacos o sustancias químicas. La información relativa a la retirada del fármaco puede no existir o no estar clara.

. Improbable

Un acontecimiento clínico, incluida una anomalía en una prueba de laboratorio, cuya relación temporal con respecto a la administración del fármaco hace improbable que exista una relación causal y para el que el uso de otros fármacos o sustancias químicas o la existencia de una enfermedad subyacente proporcionan explicaciones plausibles.

. Condicional / no clasificado

Un acontecimiento clínico, incluida una anomalía en una prueba de laboratorio, notificado como reacción adversa, para cuya adecuada evaluación son esenciales mas datos o para el que dichos datos adicionales se encuentran aun en proceso de análisis.

. Inevaluable / inclasificable

Un informe en el que se sugiere una reacción adversa que no puede valorarse debido a que la información es insuficiente o contradictoria y no puede complementarse ni verificarse.

. No relacionado

Existe suficiente información disponible para constatar que la etiología no guarda relación con la medicación del estudio.

Las medidas adoptadas al inicio de los acontecimientos adversos se clasifican y describen de la siguiente forma:

1) Ninguna, es decir, no se realizaron cambios en la medicación del estudio

2) Se retiró la medicación del estudio

3) El acontecimiento/síntoma adverso se trató de forma diferente / médicamente

4) Otras medidas (texto claro)

El curso y la evolución del acontecimiento adverso se comentarán de la siguiente manera:

1) Recuperado / resuelto

2) En fase de recuperación / resolución

3) No recuperado / no resuelto

4) Recuperado / resuelto con secuelas

5) Mortal

6) Desconocidos

Todo paciente que sufra un AA al final del estudio tiene que recibir seguimiento hasta la resolución del AA o durante un máximo de 4 semanas tras la finalización de la participación del paciente en el estudio. Una vez transcurridas 4 semanas, el investigador deberá emitir una declaración final sobre la evolución del AA.

9.2.2 Documentación y notificación de los acontecimientos adversos serios

En el momento de su incorporación al estudio, se indicará a los pacientes que deben ponerse en contacto con el investigador en caso de que se produzca un AA serio o inesperado, de modo que puedan adoptarse las medidas adecuadas.

Todo AAS (incluida la muerte, independientemente de su causa) ocurrido durante o hasta 14 días después de la finalización del estudio debe notificarse sin demora, es decir, en un plazo máximo de 24 horas, por fax al director del estudio, independientemente de su relación con la administración de la medicación del estudio (información mínima exigida: nombre del investigador / centro de estudio, número del paciente, iniciales del paciente, fecha de la primera dosis, fecha de la ultima dosis, fecha del acontecimiento, descripción del acontecimiento, evaluación de la causalidad y medidas adoptadas para contrarrestarlo).

Se facilitará a los investigadores un formulario de documentación especifico para los AAS. En caso de ocurrir un AAS, el medico deberá completar este formulario en calidad de informe inicial y enviarlo (por fax) al director del proyecto. Si es preciso, deberá rellenarse un informe de seguimiento en el que se incluirá toda la nueva información obtenida acerca del AAS, para lo que se utilizara una versión actualizada de este ‘formulario de AAS’, que se enviará también al director del proyecto.

El informe debe contener una descripción detallada de los síntomas observados y del tratamiento concomitante administrado. Además, el investigador debe comentar si existe una posible relación causal entre el AAS y la medicación del ensayo. Deberá realizarse un seguimiento de cada AAS hasta que este se resuelva o pueda explicarse satisfactoriamente. Deberá aplicarse sin reservas el procedimiento general para la observación, la recogida y el análisis de los riesgos farmacológicos (asuntos en materia de regulación) de conformidad con la Ley del Medicamento nacional.

El promotor solo desenmascarará el tratamiento de casos aislados si ello reviste importancia para la seguridad del paciente participante en el estudio. En caso de producirse un AAS intolerable, debe retirarse al paciente del ensayo clínico, cuando el investigador así lo decida, y administrase tratamiento sintomático. Las medidas adoptadas deben registrarse en el CRD.

**10– ASPECTOS ETICOS**

Este estudio se realizará de conformidad con la Declaración de Helsinki (en su última versión pertinente), los estándares internacionales y la reglamentación nacional respectiva (ver sección 10.3).

**10.1- INFORMACIÓN PARA EL PACIENTE Y CONSENTIMIENTO DEL PACIENTE**

Antes de su inclusión en el ensayo clínico, se informará a cada paciente de que su participación en este ensayo es voluntaria y de que puede retirarse del estudio en cualquier momento sin tener que explicar sus motivos y sin temor a sufrir ningún tipo de pérdida en su atención médica.

El paciente recibirá información acerca de la terapia del estudio y de sus posibles efectos adversos. Al mismo tiempo, se le explicarán el objetivo y la significación del estudio. La explicación también incluirá información acerca de su protección mediante un seguro y de sus obligaciones como asegurado.

El paciente tendrá tiempo y oportunidades suficientes para aclarar toda duda no resuelta. Además, se le hará entrega de un “Formulario de información para el paciente” que contendrá toda la información importante por escrito. En el Apéndice 4 se incluye una muestra de la información para el paciente y del consentimiento informado.

El consentimiento del paciente debe obtenerse por escrito antes del inicio del estudio. Al firmar el consentimiento informado, el paciente declara que participa voluntariamente en el ensayo y que tiene la intención de seguir las instrucciones del protocolo del estudio y las instrucciones del investigador, así como de responder a las preguntas que se le hagan durante el transcurso del ensayo. Se informará a cada paciente de que su participación en este ensayo es voluntaria y de que puede retirarse del estudio en cualquier momento sin tener que explicar sus motivos. El investigador guardará una copia del consentimiento informado por escrito del paciente en el lugar designado a tal efecto en el archivo del investigador.

**10.2 - COMITÉ DE ÉTICA**

De conformidad con la normativa nacional antes del inicio del estudio se procederá a enviar al Comité de Ética el protocolo del estudio y la información para el paciente / consentimiento informado. Se obrará conforme al criterio de asesoramiento de dicho comité.

**10.3 – POLIZA DE SEGURO**

En el presente estudio se investiga el efecto de una dieta sin gluten en comparación con una dieta con gluten (que contiene la cantidad de gluten que lleva una dieta estándar). La dieta sin gluten es bien tolerada, contiene los macronutrientes y micronutrientes indispensables para la nutrición adecuada, y no se asocia a efectos secundarios.

Ya que no se investiga el efecto de un fármaco y que la dieta sin gluten no produce efectos secundarios, y después del asesoramiento legal correspondiente, se considera que no es necesario concertar ninguna póliza de seguro de responsabilidad civil.

**10.4 - BASES LEGALES**

El estudio debe realizarse de conformidad con el protocolo, con las normas de BPC y con los requisitos reglamentarios pertinentes.

**10.4.1 - Declaración de Helsinki**

El estudio se llevará a cabo de conformidad con los “Principios éticos para la investigación médica con seres humanos” de la 18ª Asamblea General de la Asociación Médica Mundial(AMM) en Helsinki (1964), de sus enmiendas, efectuadas por las 29ª, 35ª, 41ª, 48ª y 52ª Asambleas Generales de la AMM (Tokyo 1975, Venecia 1983, Hong Kong 1989, Somerset West 1996 y Edimburgo 2000), y de la Nota de Clarificación sobre el Párrafo 29 añadida por la Asamblea General de la AMM en Washington 2002 y la Nota de Clarificación sobre el Párrafo 30 añadida por la Asamblea General de la AMM en Tokyo 2004.

**10.4.2 - Otras bases legales**

Las otras bases legales de este ensayo clínico son las siguientes:

• Tema E6 de la ICH, Guías para las Buenas Prácticas Clínicas, incluida la errata posterior al paso 4, septiembre de 1997

• Directiva 2001/20/CE (4 de abril de 2001)

• Directiva de la Comisión 2005/28/CE (8 de abril de 2005)

• Requisitos reglamentarios nacionales / guías de los países participantes en torno a los ensayos clínicos.

Se hará entrega al experto médico y a todos los investigadores de un folleto del investigador actualizado en el que se describirá con todo detalle el estado de los conocimientos preclínicos y clínicos sobre la medicación del estudio. Tan pronto como se obtenga nueva información, se facilitará una versión actualizada o se añadirá una enmienda al folleto del investigador preexistente.

**10.4.3 - Aprobación de las autoridades reguladoras**

No puede incluirse a ningún paciente en el estudio antes de que se hayan cumplido los requisitos respectivos de las autoridades sanitarias nacionales. El ensayo comenzará sólo tras la aprobación del Comité de Ética responsable y tras la conformidad de parte de la autoridad sanitaria nacional correspondiente.

**11 - RESPONSABILIDADES**

El promotor, los investigadores y todo el personal involucrado en el estudio acuerdan realizar este ensayo clínico de conformidad con las Guías de la ICH para las Buenas Prácticas Clínicas.

De acuerdo con la legislación del Estado (R.D. 223/2004) y de la Directiva Comunitaria (91/507/CEE), todos los ensayos clínicos en todas sus fases, incluyendo aquellos de biodisponibilidad y bioequivalencia, se realizarán según las normas de buena práctica clínica.

Las normas de buena práctica clínica (BPC) son una ayuda para asegurar que la investigación clínica se lleva a cabo según los más elevados estándares de calidad, siguiendo los criterios éticos (basados en la Declaración de Helsinki y desarrollos ulteriores) y con un equipo médico de garantía. En este sentido, las normas de BPC constituyen una norma para el diseño, dirección, realización, cumplimiento, monitorización, auditoría, registro, análisis e información de ensayos clínicos que asegura que los datos y resultados obtenidos son correctos y creíbles y que se protegen los derechos, integridad y confidencialidad de los sujetos del ensayo.

Los principios de la BPC (buena práctica clínica) son los siguientes:

1. Los ensayos clínicos deben ser realizados de acuerdo con los principios éticos que tienen su origen en la Declaración Helsinki, los cuales son consistentes con la BPC y los requisitos reguladores pertinentes.
2. Antes de iniciar un ensayo, deben considerarse las inconveniencias y riesgos previsibles en relación con el beneficio previsto paras el sujeto individual del ensayo y para la sociedad. Un ensayo debe ser iniciado y continuado sólo si los beneficios previstos justifican los riesgos.
3. Los derechos, seguridad y bienestar de los sujetos del ensayo son las consideraciones más importantes y deben prevalecer sobre los intereses de la ciencia y la sociedad.
4. La información clínica y no clínica disponible sobre un producto en investigación debe ser adecuada para respaldar el ensayo clínico propuesto.
5. Los ensayos clínicos deben ser científicamente razonables y estar descritos en un protocolo claro y detallado.
6. Un ensayo debe ser realizado de acuerdo con el protocolo, que ha recibido una revisión previa y una opinión favorable/aprobación de un Consejo Institucional de Revisión (CIR) / Comité Ético Independiente (CEI).
7. El cuidado médico que reciben los sujetos y las decisiones médicas que afectan a los mismos deben ser siempre responsabilidad de un médico cualificado o, en su caso de un odontólogo cualificado.
8. Cada individuo implicado en la realización de un ensayo debe ser cualificado, por educación, formación y experiencia, para realizar su labor.
9. Se debe obtener un consentimiento informado, dado libremente, de cada sujeto, previamente a su participación en el ensayo clínico.
10. Toda la información del ensayo clínico debe ser registrada, manejada y almacenada de forma que permita su comunicación, verificación e interpretación exactas.
11. Debe protegerse la confidencialidad de los registros que pudieran identificar a los sujetos, respetando la privacidad y las normas de confidencialidad de acuerdo con los requisitos reguladores pertinentes.
12. Los productos en investigación deben ser fabricados, manejos y almacenados de acuerdo con las normas de buena práctica de fabricación pertinentes y se deben utilizar de acuerdo con el protocolo aprobado.

Se llevarán a cabo los sistemas y procedimientos que aseguren la calidad de cada aspecto del ensayo.

**11.1 Acceso directo a los documentos fuente conforme a las guías BPC ICH**

De acuerdo con las BPC ICH, los investigadores / centros deben facilitar el acceso directo a los datos / documentos fuente para permitir la realización de las actividades relacionadas con el ensayo de monitorización, auditoria, revisión por parte del Comité de Ética Independiente (CEI) e inspección reglamentaria. Todos los participantes han consentido (a través del consentimiento informado por escrito) al acceso directo a sus registros médicos originales para las actividades relacionadas con el ensayo de monitorización, revisión por parte del CEI e inspección reglamentaria. El contenido del protocolo debe incluir la identificación de todo dato que se deba registrar directamente en los CRD (es decir, sin registro escrito o electrónico previo de los datos) y que se deba considerar dato fuente.

En el archivo del paciente se deben registrar al menos los siguientes datos:

• datos demográficos del paciente (nombre, dirección, fecha de nacimiento, sexo)

• estatura y peso (basales).

• enfermedad del estudio subyacente, fecha de los primeros síntomas y fecha en la que se diagnosticó por primera vez la enfermedad del estudio.

• resultados de las endoscopias y los exámenes histológicos previos, cuando corresponda.

• medicaciones previas en relación con la enfermedad del estudio.

• enfermedades y medicaciones concomitantes.

• constancia de que el paciente está participando en este ensayo.

• fecha de cada visita.

• resultados de las exploraciones efectuadas en este ensayo.

• actividad de la enfermedad del estudio / estado general del paciente en cada visita.

• todos los hallazgos de laboratorio generados en este ensayo.

• acontecimientos adversos o reacciones medicamentosas adversas.

• motivos para la suspensión, cuando corresponda.

Todas las demás variables pueden introducirse directamente en el CRD y se consideran datos fuente.

**11.2 Archivado de los documentos esenciales**

11.2.1 Investigador

El investigador debe guardar sus copias de los CRD, los consentimientos informados por escrito, la lista de identificación de los pacientes y los registros de los pacientes con los datos originales durante un mínimo de 15 años.

11.2.2 Promotor

El promotor guardará todos los CRD y toda la documentación adicional relativa al

ensayo durante un mínimo de 15 años.

**12. CONSIDERACIONES ADICIONALES**

**12.1.- CONDICIONES DE PUBLICACIÓN**

Al finalizar el estudio se comunicarán los resultados a los Comités Éticos de Investigación y a la Agencia Española del Medicamento. Los resultados serán propiedad del promotor.

Todas las publicaciones que surjan del estudio irán firmadas por todos los Investigadores Principales. En los centros en que se incluyan más de 10 pacientes podrá firmar un colaborador adicional.

**12.2.- PROCEDIMIENTO DE ENMIENDA DEL PROTOCOLO**

Cualquier modificación del presente protocolo que no implique un cambio sustancial podrá efectuarse de común acuerdo, siempre por escrito, entre el investigador principal y el promotor.

En caso de que las modificaciones puedan ocasionar cambios sustanciales en el protocolo original deberá someterse a consideración del Comité Ético de Investigación y notificarse a la Agencia Española del Medicamento.

**12.3.- ANULACIÓN DEL ENSAYO**

En caso de anularse el ensayo se informará a los investigadores, a los CEIC de los hospitales y a la Agencia Española del Medicamento sobre las razones que han aconsejado la suspensión/anulación del mismo.

**13- ANÁLISIS ESTADÍSTICO**

El cálculo del tamaño de la muestra se basa en la variable principal de valoración de la eficacia, es decir, de la tasa de remisión a los 6 meses. En base a los resultados de estudios previos se considera que la tasa de remisión con una dieta sin gluten será del 70% y con placebo del 20%. Asumiendo un 10% de pérdidas de pacientes, un valor alfa del 5% y una potencia estadística (valor beta) del 80%, para una prueba bilateral, se ha calculado que son necesarios 20 pacientes en cada grupo terapéutico (teniendo en cuenta que para analizar los resultados se necesitará una prueba de Chi-cuadrado con corrección de Yates). La N total será, pues, de 40 pacientes.

El análisis estadístico se realizará, en primer lugar, por intención de tratamiento (IT). Los sujetos se incluirán en el análisis IT si han tomado la medicación al menos una dosis de la medicación del estudio.

El análisis por protocolo (PP) incluirá a los pacientes que hayan tomado la medicación al menos durante 30 días, y de los que se disponga información sobre la variable principal de eficacia a los 30 días. Quedarán excluidos de este análisis aquellos sujetos que presenten desviaciones mayores del protocolo.

Todas las evaluaciones de las variables principales y secundarias, tanto de eficacia como de seguridad, se entenderán en sentido exploratorio (alfa=0,05, bilateral).

Se utilizará la prueba de Chi cuadrado para grupos independientes con correción de Yates o el test exacto de Fisher para las variables cualitativas. Para la comparaciones intergrupos de las variables cuantitativas se empleará el test de la t de Student para las variables paramétricas, y el test de la U de Mann-Whitney para las variables no paramétricas.

Se considerarán diferencias significativas el hallazgo de un valor de p inferior a 0,05. Se proporcionará la diferencia entre las tasas de remisión clínica entre los grupos de tratamiento y el intervalo de confianza del 95% bilateral correspondiente, como estimación del efecto terapéutico.

**14.- BIBLIOGRAFIA**

1. Di Sabatino A, Corazza GR. Coeliac disease. Lancet 2009; 373:1480-93.
2. Troncone R, Jabri B. Coeliac disease and gluten sensitivity. J Intern Med 2011;269:582-90.
3. Bizzaro N, Tozzoli R, Villalta D Fabris M, Tonutti E. Cutting-edge issues in celiac disease and in gluten intolerance. Clinic Rev Allerg Immunol 2010; Dec 23.
4. Biesiekierski JR, Newnham de, Irving PM, Barrett JS, et al. Gluten causes gastrointestinal symptoms in subjects without celiac disease: A double-blind randomized placebo-controlled trial. Am J Gastroenterol 2011 (online only,11 January 2011)

**APENDICE 1**

**GLUTEN 10 g MONODOSIS: MEMÒRIA TÈCNICA D’ELABORACIÓ**

**FARMACIA XALABARDER (Pujades 247, Barcelona)**

1. **DESCRIPCIÓ DEL PRODUCTE**

Gluten 10g Monodosis

1. **COMPOSICIÓ**

Aquest producte es troba compost de 10g de gluten en cada unitat.

1. **PRESENTACIÓ**

Sobres minodosis de 10g de producte.

Caixes de 60 sobres.

6 caixes per cada tractament

1. **MATÈRIES PRIMERES**

La matèria primera es troba adquirida a l’empresa Fagron Ibèrica.

El certificat d’anàlisi es troba compost dels següents assajos i resultats:

|  | **GLUTEN** | |
| --- | --- | --- |
| **ASSAJOS** | | **RESULTATS** |
| **PÈRDUDA PER DESSECACIÓ** | | 7,8% |
| **pH** | | <5 |
| **CENDRES TOTALS** | | 0,9% |
| **PROTEÏNES** | | 82 |
| **FIBRA** | | 0,5 |
| **MATÈRIA GREIXOSA** | | 1,3 |
| **CARBOHIDRATS** | | 9 |
|  | | **COMPLEIX ESPECIFICACIONS** |

1. **MATERIAL ACONDICIONAMENT**

Blister d’alumini i cel·lulosa estucada tipus “Surling”, tancat per segellament tèrmic.

Els sobres van identificats amb el número de referència de l’elaboració i la data de caducitat. S’ha demanat expressament que no porti la composició escrita.

Les caixes van identificades amb la següent etiqueta:

1. **CADUCITAT**

Data de caducitat del producte: 29/5/2012

1. **ELABORACIÓ**

Data d’elaboració: 29/5/2009

Referència d’elaboració: 820055963

Acceptació: S’ha elaborat segons els estàndards d’elaboració de pólvores per ús oral en monodosis. Ha passat els controls de qualitat realitzats. El producte es considera ACCEPTAT.

1. **INDICACIÓ**

S’ha sol·licitat el producte des del Servei de Farmàcia de la Mútua de Terrassa per a un assaig clínic de codi EL-DSG/01/01: gluten /maltodextrina.

1. **SIGNATURA**

| **Director tècnic** | **Unitat de Garantia de Qualitat** |
| --- | --- |
| Eduard Xalabarder | Roser Masanas |

**APÉNDICE 2**

**MEMORIA TECNICA MALTODEXTRINA: 10 G MONODOSIS**

**Farmàcia Xalabarder (Pujades 247, Barcelona)**

1. **DESCRIPCIÓ DEL PRODUCTE**

Maltodextrina 10g Monodosis

1. **COMPOSICIÓ**

Aquest producte es troba compost de 10g de maltodextrina en cada unitat.

1. **PRESENTACIÓ**

Sobres minodosis de 10g de producte.

Caixes de 60 sobres.

6 caixes per cada tractament

1. **MATÈRIES PRIMERES**

La matèria primera es troba adquirida a l’empresa Fagron Ibèrica.

El certificat d’anàlisi es troba compost dels següents assajos i resultats:

|  | **MALTODEXTRINA** | |
| --- | --- | --- |
| **ASSAJOS** | | RESULTATS |
| **IDENTIFICACIÓN** | | CONFORME |
| **PÈRDUDA PER DESSECACIÓ** | | 4,74% |
| **METALLS PESATS** | | <10ppm |
| **CENDRES SULFÚRIQUES** | | <0,5% |
| **pH** | | 6,02 |
| **DIÒXID DE SOFRE** | | <5ppm |
| **AEROBIS TOTALS** | | <10ufc/g |
| **FONGS I LLEVATS** | | <10ufc/g |
| **E. COLI** | | CONFORME |
| **SALMONELLA** | | CONFORME |
| **DEXTROSA** | | CONFORME |
|  | | **COMPLEIX ESPECIFICACIONS** |

1. **MATERIAL ACONDICIONAMENT**

Blister d’alumini i cel·lulosa estucada tipus “Surling”, tancat per segellament tèrmic.

Els sobres van identificats amb el número de referència de l’elaboració i la data de caducitat. S’ha demanat expressament que no porti la composició escrita.

Les caixes van identificades amb la següent etiqueta:

1. **CADUCITAT**

Data de caducitat del producte: 29/5/2012

1. **ELABORACIÓ**

Data d’elaboració: 29/5/2009

Referència d’elaboració: 820055964

Acceptació: S’ha elaborat segons els estàndards d’elaboració de pólvores per ús oral en monodosis. Ha passat els controls de qualitat realitzats. El producte es considera ACCEPTAT.

1. **INDICACIÓ**

S’ha sol·licitat el producte des del Servei de Farmàcia de la Mútua de Terrassa per a un assaig clínic de codi EL DSG/01/01: gluten /maltodextrina.

1. **SIGNATURA**

| **Director tècnic** | **Unitat de Garantia de Qualitat** |
| --- | --- |
| Eduard Xalabarder | Roser Masanas |

**APÉNDICE 3**

**Calidad de vida del paciente**

El paciente deberá contestar a un total de 36 preguntas (elementos) relativas a los efectos de la enfermedad sobre su calidad de vida (CV). Cada elemento se puntúa de acuerdo con una escala graduada de 5 puntos, desde el 1 (que indica una mala CV) hasta el 5 (que indica una buena CV); por consiguiente, la puntuación global del GIQLI oscilará entre 0 y 180.

Para facilitar la interpretación, la suma de cada una de las respuestas de cada escala (síntomas, bienestar físico, bienestar emocional, y relaciones sociales), dividida por el número de preguntas en la escala, proporciona la puntación de la escala. La puntuación global del GIQLI es la suma de las puntuaciones de todas las escalas.

Las preguntas y su pertenencia a cada una de las cuatro escalas son las siguientes:

**CUESTIONARIO DE CALIDAD DE VIDA GASTROINTESTINAL (GIQLI)**

**1. Durante las 2 últimas semanas ¿ha sentido dolor de estómago o de tripa?**

1. *Todo el tiempo* **2.** *Casi todo el tiempo* **3.** *algunas veces* **4.** *raramente* **5.** *nunca.*

*(Síntomas)*

**2. Durante las 2 últimas semanas ¿Ha sentido sensación de plenitud abdominal (o de tripa llena)?**

1. *Todo el tiempo* **2.** *Casi todo el tiempo* **3.** *algunas veces* **4.** *raramente* **5.** *nunca.*

*(Síntomas)*

**3. Durante las 2 últimas semanas ¿ha sentido hinchazón (sensación de tener muchos gases en el estómago)?**

1. *Todo el tiempo* **2.** *Casi todo el tiempo* **3.** *algunas veces* **4.** *raramente* **5.** *nunca.*

*(Síntomas)*

**4. Durante las 2 últimas semanas ¿ha sentido escape de ventosidades?**

1. *Todo el tiempo* **2.** *Casi todo el tiempo* **3.** *algunas veces* **4.** *raramente* **5.** *nunca.*

*(Síntomas)*

**5. Durante las 2 últimas semanas ¿ha sentido fuertes eructos?**

1. *Todo el tiempo* **2.** *Casi todo el tiempo* **3.** *algunas veces* **4.** *raramente* **5.** *nunca.*

*(Síntomas)*

**6. Durante las 2 últimas semanas ¿ha sentido ruidos llamativos en el estómago o las tripas?**

1. *Todo el tiempo* **2.** *Casi todo el tiempo* **3.** *algunas veces* **4.** *raramente* **5.** *nunca.*

*(Síntomas)*

**7. Durante las 2 últimas semanas ¿ha sentido la necesidad de hacer de vientre con mucha frecuencia?**

1. *Todo el tiempo* **2.** *Casi todo el tiempo* **3.** *algunas veces* **4.** *raramente* **5.** *nunca.*

*(Síntomas)*

**8. Durante las 2 últimas semanas ¿ha gozado o ha sentido placer comiendo?**

1. *Todo el tiempo* **2.** *Casi todo el tiempo* **3.** *algunas veces* **4.** *raramente* **5.** *nunca.*

*(Síntomas)*

**9. Durante las 2 últimas semanas ¿con qué frecuencia ha renunciado usted a comidas que le gustan como consecuencia de su estado de salud actual?**

1. *Muchísimo* **2.** *mucho* **3.** *algo* **4.** *un poco* **5.** *nada.*

*(Síntomas)*

**10. Durante las 2 últimas semanas ¿cómo ha sobrellevado las tensiones diarias?**

1. *Muy mal* **2.** *mal* **3.** *regular* **4.** *bien* **5.** *muy bien.*

*(Emocional)*

**11. Durante las 2 últimas semanas ¿se ha sentido triste o deprimido?**

1. *Todo el tiempo* **2.** *Casi todo el tiempo* **3.** *algunas veces* **4.** *raramente* **5.** *nunca.*

*(Emocional)*

**12. Durante las 2 últimas semanas ¿se ha sentido nervioso o con miedo con motivo de su enfermedad?**

1. *Todo el tiempo* **2.** *Casi todo el tiempo* **3.** *algunas veces* **4.** *raramente* **5.** *nunca.*

*(Emocional)*

**13. Durante las 2 últimas semanas ¿se ha sentido satisfecho con su vida en general?**

1. *Todo el tiempo* **2.** *Casi todo el tiempo* **3.** *algunas veces* **4.** *raramente* **5.** *nunca.*

*(Emocional)*

**14. Durante las 2 últimas semanas ¿se ha sentido frustrado?**

1. *Todo el tiempo* **2.** *Casi todo el tiempo* **3.** *algunas veces* **4.** *raramente* **5.** *nunca.*

*(Emocional)*

**15. Durante las 2 últimas semanas ¿se ha sentido cansado o fatigado?**

1. *Todo el tiempo* **2.** *Casi todo el tiempo* **3.** *algunas veces* **4.** *raramente* **5.** *nunca.*

*(Física)*

**16. Durante las 2 últimas semanas ¿se ha sentido indispuesto?**

1. *Todo el tiempo* **2.** *Casi todo el tiempo* **3.** *algunas veces* **4.** *raramente* **5.** *nunca.*

*(Física)*

**17. Durante la última semana (¡sólo una semana!) ¿se ha despertado por la noche?**

1. *Cada noche* **2.** *5 ó 6 noches* **3.** *3 ó 4 noches* **4.** *1 ó 2 noches* **5.** *nunca.*

*(Física)*

**18. ¿En qué medida le ha provocado su estado de salud actual cambios molestos en su apariencia o aspecto físico?**

1. *Mucho* **2.** *bastante* **3.** *algo* **4.** *un poco* **5.** *nada.*

*(Física)*

**19. Como consecuencia de su estado de salud actual, ¿en qué medida ha empeorado su vitalidad?**

1. *Mucho* **2.** *bastante* **3.** *algo* **4.** *un poco* **5.** *nada.*

*(Física)*

**20. Como consecuencia de su estado de salud actual, ¿en qué medida ha perdido su aguante o resistencia física?**

1. *Mucho* **2.** *bastante* **3.** *algo* **4.** *un poco* **5.** *nada.*

*(Física)*

**21. Como consecuencia de su estado de salud actual, ¿en qué medida ha sentido disminuida su forma física?**

1. *Mucho* **2.** *bastante* **3.** *algo* **4.** *un poco* **5.** *nada.*

*(Física)*

**22. Durante las 2 últimas semanas, ¿se ha sentido contrariado o molesto por el tratamiento médico de su estado de salud actual?**

1. *Muchísimo* **2.** *mucho* **3.** *algo* **4.** *un poco* **5.** *nada.*

*(Tratamiento médico)*

**23. Durante las 2 últimas semanas, ¿ha podido llevar a cabo sus actividades cotidianas (por ejemplo, estudios, trabajo, labores domésticas)?**

1. *Todo el tiempo* **2.** *Casi todo el tiempo* **3.** *algunas veces* **4.** *raramente* **5.** *nunca.*

*(Social)*

**24. Durante las 2 últimas semanas, ¿se ha sentido capaz de participar en actividades de recreo y tiempo libre?**

1. *Todo el tiempo* **2.** *Casi todo el tiempo* **3.** *algunas veces* **4.** *raramente* **5.** *nunca.*

*(Social)*

**25. ¿En qué medida se han alterado las relaciones con sus personas cercanas (familia o amigos) como consecuencia de su estado de salud actual?**

1. *Muchísimo* **2.** *mucho* **3.** *algo* **4.** *un poco* **5.** *nada.*

*(Social)*

**26. ¿En qué medida ha resultado perjudicada su vida sexual como consecuencia de su estado de salud actual?**

1. *Muchísimo* **2.** *mucho* **3.** *algo* **4.** *un poco* **5.** *nada.*

*(Social)*

**27. Durante las ‘2 últimas semanas, ¿ha sentido que la comida o los líquidos le subían a la boca (regurgitación) ?**

1. *Todo el tiempo* **2.** *Casi todo el tiempo* **3.** *algunas veces* **4.** *raramente* **5.** *nunca.*

*(Síntomas)*

**28. Durante las 2 últimas semanas ¿ha sentido incomodidad por comer lento o despacio?**

1. *Todo el tiempo* **2.** *Casi todo el tiempo* **3.** *algunas veces* **4.** *raramente* **5.** *nunca.*

*(Síntomas)*

**29. Durante las 2 últimas semanas ¿ha tenido problemas para tragar la comida?**

1. *Todo el tiempo* **2.** *Casi todo el tiempo* **3.** *algunas veces* **4.** *raramente* **5.** *nunca.*

*(Síntomas)*

**30. Durante las 2 últimas semanas, ¿ha sentido la necesidad de tener que hacer de vientre con urgencia, de repente?**

1. *Todo el tiempo* **2.** *Casi todo el tiempo* **3.** *algunas veces* **4.** *raramente* **5.** *nunca.*

*(Síntomas)*

**31. Durante las 2 últimas semanas, ¿ha tenido diarrea?**

1. *Todo el tiempo* **2.** *Casi todo el tiempo* **3.** *algunas veces* **4.** *raramente* **5.** *nunca.*

*(Síntomas)*

**32. Durante las 2 últimas semanas, ¿ha tenido estreñimiento?**

1. *Todo el tiempo* **2.** *Casi todo el tiempo* **3.** *algunas veces* **4.** *raramente* **5.** *nunca.*

*(Síntomas)*

**33. Durante las 2 últimas semanas, ¿ha tenido náuseas?**

1. *Todo el tiempo* **2.** *Casi todo el tiempo* **3.** *algunas veces* **4.** *raramente* **5.** *nunca.*

*(Síntomas)*

**34. Durante las 2 últimas semanas, ¿ha tenido sangre en las heces?**

1. *Todo el tiempo* **2.** *Casi todo el tiempo* **3.** *algunas veces* **4.** *raramente* **5.** *nunca.*

*(Síntomas)*

**35. Durante las 2 últimas semanas, ¿ha tenido acidez de estómago?**

1. *Todo el tiempo* **2.** *Casi todo el tiempo* **3.** *algunas veces* **4.** *raramente* **5.** *nunca.*

*(Síntomas)*

**36. Durante las 2 últimas semanas, ¿ha tenido problemas para contener las heces?**

1. *Todo el tiempo* **2.** *Casi todo el tiempo* **3.** *algunas veces* **4.** *raramente* **5.** *nunca.*

*(Síntomas)*

**APENDICE 4**

**Registro del consumo de alimentos de tres días**

**Instrucciones**

- En este cuestionario deberá ir anotando todos los alimentos y bebidas consumidos durante tres días, incluyendo un festivo.
- Es muy importante no cambiar el régimen habitual de comidas.
- Para evitar que se olvide algún alimento, conviene anotar todo inmediatamente después de

comer. No olvide indicar todos los ingredientes de cada receta.

- También deberá anotar todas las comidas realizadas fuera de casa.
- El cuestionario consta de dos hojas para cada día. En la primera deberá anotar todos los menús y procesos culinarios y en la segunda tendrá que describir con detalle todos los ingredientes y cantidades (pesando o mediante medidas caseras: cucharada sopera, de postre, vaso de agua, vino, plato hondo, .......). Trate de estimar el aceite en cucharadas soperas o de postre.
- Indique si el peso del alimento se refiere al alimento crudo o cocinado, con o sin desperdicios.
- Cada hoja deberá estar identificada con la fecha y el día de la semana.
- En la parte posterior de la hoja, anote las recetas de los platos muy elaborados.
- No olvide indicar: azúcar, pan, aceite, tapas, refrescos, bebidas alcohólicas, dulces, chocolate, frutos secos, patatas fritas, ...
- En cuanto a la descripción de los alimentos, es importante mencionar la calidad y tipo del

alimento: tipo de leche (entera, semidesnatada o desnatada), tipo de carnes y pescados, tipo de pan (pan blanco o integral), mantequilla o margarina, etc.

- Siempre que sepa el nombre comercial del producto, anótelo.
- Anotar si el producto comercial está etiquetado como “Sin Gluten”.
- Anote el tipo y marca del aceite/s utilizado/s.
- Indique si se trata de un alimento precocinado, listo para comer, …
- Anote todas las dudas que le hayan surgido al rellenar el cuestionario.

**Hoja de Menús PRIMER DÍA**

Nombre: Nº Código:

Fecha: Edad: Sexo:

Día de la semana: Hospital (iniciales):

| Hora:  Lugar: | DESAYUNO |
| --- | --- |
| Hora:  Lugar: | MEDIA MAÑANA |
| Hora:  Lugar: | COMIDA |
| Hora:  Lugar: | MERIENDA |
| Hora:  Lugar: | CENA |
| Hora:  Lugar: | OTRAS |

**PRIMER DÍA: INGREDIENTES Y CANTIDADES**

| **ALIMENTO (Marca comercial si existe)** | **CANTIDAD** | **¿Etiquetado como “Sin Gluten”?** |
| --- | --- | --- |
|  |  |  |
|  |  |  |
|  |  |  |
|  |  |  |
|  |  |  |
|  |  |  |
|  |  |  |
|  |  |  |
|  |  |  |
|  |  |  |
|  |  |  |
|  |  |  |
|  |  |  |
|  |  |  |
|  |  |  |
|  |  |  |
|  |  |  |
|  |  |  |
|  |  |  |
|  |  |  |
|  |  |  |
|  |  |  |
|  |  |  |
|  |  |  |
|  |  |  |
|  |  |  |
|  |  |  |
|  |  |  |
|  |  |  |
|  |  |  |
|  |  |  |
|  |  |  |
|  |  |  |
|  |  |  |
|  |  |  |
|  |  |  |
|  |  |  |
|  |  |  |
|  |  |  |
|  |  |  |
|  |  |  |
|  |  |  |
| **ALIMENTO (Marca comercial si existe)** | **CANTIDAD** | **¿Etiquetado como “Sin Gluten”?** |
|  |  |  |
|  |  |  |
|  |  |  |
|  |  |  |
|  |  |  |
|  |  |  |
|  |  |  |
|  |  |  |
|  |  |  |
|  |  |  |
|  |  |  |
|  |  |  |
|  |  |  |
|  |  |  |
|  |  |  |
|  |  |  |
|  |  |  |
|  |  |  |
|  |  |  |
|  |  |  |
|  |  |  |
|  |  |  |
|  |  |  |
|  |  |  |
|  |  |  |
|  |  |  |
|  |  |  |
|  |  |  |
|  |  |  |
|  |  |  |
|  |  |  |
|  |  |  |
|  |  |  |
|  |  |  |
|  |  |  |
|  |  |  |
|  |  |  |
|  |  |  |
|  |  |  |
|  |  |  |
|  |  |  |
|  |  |  |
|  |  |  |

**Hoja de Menús SEGUNDO DÍA**

Fecha:

Día de la semana:

| Hora:  Lugar: | DESAYUNO |
| --- | --- |
| Hora:  Lugar: | MEDIA MAÑANA |
| Hora:  Lugar: | COMIDA |
| Hora:  Lugar: | MERIENDA |
| Hora:  Lugar: | CENA |
| Hora:  Lugar: | OTRAS |

**SEGUNDO DÍA: INGREDIENTES Y CANTIDADES**

| **ALIMENTO (Marca comercial si existe)** | **CANTIDAD** | **¿Etiquetado como “Sin Gluten”?** |
| --- | --- | --- |
|  |  |  |
|  |  |  |
|  |  |  |
|  |  |  |
|  |  |  |
|  |  |  |
|  |  |  |
|  |  |  |
|  |  |  |
|  |  |  |
|  |  |  |
|  |  |  |
|  |  |  |
|  |  |  |
|  |  |  |
|  |  |  |
|  |  |  |
|  |  |  |
|  |  |  |
|  |  |  |
|  |  |  |
|  |  |  |
|  |  |  |
|  |  |  |
|  |  |  |
|  |  |  |
|  |  |  |
|  |  |  |
|  |  |  |
|  |  |  |
|  |  |  |
|  |  |  |
|  |  |  |
|  |  |  |
|  |  |  |
|  |  |  |
|  |  |  |
|  |  |  |
|  |  |  |
|  |  |  |
|  |  |  |
|  |  |  |
| **ALIMENTO (Marca comercial si existe)** | **CANTIDAD** | **¿Etiquetado como “Sin Gluten”?** |
|  |  |  |
|  |  |  |
|  |  |  |
|  |  |  |
|  |  |  |
|  |  |  |
|  |  |  |
|  |  |  |
|  |  |  |
|  |  |  |
|  |  |  |
|  |  |  |
|  |  |  |
|  |  |  |
|  |  |  |
|  |  |  |
|  |  |  |
|  |  |  |
|  |  |  |
|  |  |  |
|  |  |  |
|  |  |  |
|  |  |  |
|  |  |  |
|  |  |  |
|  |  |  |
|  |  |  |
|  |  |  |
|  |  |  |
|  |  |  |
|  |  |  |
|  |  |  |
|  |  |  |
|  |  |  |
|  |  |  |
|  |  |  |
|  |  |  |
|  |  |  |
|  |  |  |
|  |  |  |
|  |  |  |
|  |  |  |
|  |  |  |

**Hoja de Menús TERCER DÍA**

Fecha:

Día de la semana:

| Hora:  Lugar: | DESAYUNO |
| --- | --- |
| Hora:  Lugar: | MEDIA MAÑANA |
| Hora:  Lugar: | COMIDA |
| Hora:  Lugar: | MERIENDA |
| Hora:  Lugar: | CENA |
| Hora:  Lugar: | OTRAS |

**TERCER DÍA: INGREDIENTES Y CANTIDADES**

| **ALIMENTO (Marca comercial si existe)** | **CANTIDAD** | **¿Etiquetado como “Sin Gluten”?** |
| --- | --- | --- |
|  |  |  |
|  |  |  |
|  |  |  |
|  |  |  |
|  |  |  |
|  |  |  |
|  |  |  |
|  |  |  |
|  |  |  |
|  |  |  |
|  |  |  |
|  |  |  |
|  |  |  |
|  |  |  |
|  |  |  |
|  |  |  |
|  |  |  |
|  |  |  |
|  |  |  |
|  |  |  |
|  |  |  |
|  |  |  |
|  |  |  |
|  |  |  |
|  |  |  |
|  |  |  |
|  |  |  |
|  |  |  |
|  |  |  |
|  |  |  |
|  |  |  |
|  |  |  |
|  |  |  |
|  |  |  |
|  |  |  |
|  |  |  |
|  |  |  |
|  |  |  |
|  |  |  |
|  |  |  |
|  |  |  |
|  |  |  |
| **ALIMENTO (Marca comercial si existe)** | **CANTIDAD** | **¿Etiquetado como “Sin Gluten”?** |
|  |  |  |
|  |  |  |
|  |  |  |
|  |  |  |
|  |  |  |
|  |  |  |
|  |  |  |
|  |  |  |
|  |  |  |
|  |  |  |
|  |  |  |
|  |  |  |
|  |  |  |
|  |  |  |
|  |  |  |
|  |  |  |
|  |  |  |
|  |  |  |
|  |  |  |
|  |  |  |
|  |  |  |
|  |  |  |
|  |  |  |
|  |  |  |
|  |  |  |
|  |  |  |
|  |  |  |
|  |  |  |
|  |  |  |
|  |  |  |
|  |  |  |
|  |  |  |
|  |  |  |
|  |  |  |
|  |  |  |
|  |  |  |
|  |  |  |
|  |  |  |
|  |  |  |
|  |  |  |
|  |  |  |
|  |  |  |
|  |  |  |

**APENDICE 5.**

**EVALUACIÓN DEL GRADO DE ADHERENCIA A LA DIETA SIN GLUTEN REALIZADA POR UN DIETISTA EXPERTA EN ENFERMEDAD CELÍACA**

|  |  | **Siempre**  **(0)** | **Casi**  **siempre**  **(1)** | **Ocasionalmente**  **(2)** | **Casi nunca**  **(3)** | **Nunca**  **(4)** |
| --- | --- | --- | --- | --- | --- | --- |
| **1** | Utiliza restaurantes aptos para celiacos y/o hace preguntas a fondo cuando come fuera de casa |  |  |  |  |  |
| **2** | Sigue el lema “si hay duda déjalo” |  |  |  |  |  |
| **3** | Comprueba medicamentos, suplementos y productos de cuidado personal que entran por nariz, ojos y boca |  |  |  |  |  |
| **4** | Lee las etiquetas de los productos comerciales, las entiende y las comprueba |  |  |  |  |  |
| **5** | Elimina las fuentes de contaminación potencial en la cocina o en la mesa |  |  |  |  |  |
|  |  | **Siempre**  **(4)** | **Casi**  **siempre**  **(3)** | **Ocasionalmente**  **(2)** | **Casi nunca**  **(1)** | **Nunca**  **(0)** |
| **6** | Come avena (contaminada) |  |  |  |  |  |
| **7** | Consume gluten intencionadamente o no (según encuesta dietética o por estilo de vida) |  |  |  |  |  |
| **8** | Confía en su pareja o miembro de familia para realizar adecuadamente la DSG |  |  |  |  |  |

|  | **Preguntas sociales** | **Sí** | **No** |
| --- | --- | --- | --- |
| **1** | Muestra adherencia/motivación mala debido a negación, angustia, economía, problemas familiares o sociales |  |  |
| **2** | Tiene problemas mentales o de conducta que dificulten la adherencia a la dieta |  |  |
| **3** | Tiene problemas de lenguaje (idioma) que hacen más difícil la adherencia a la dieta |  |  |
| **4** | Consume alimentos con gluten por motivos religiosos (p.e., comunión, pascua judía…) |  |  |
| **5** | Requiere educación repetida sobre gluten oculto en dieta |  |  |

**Excelente** : 0 a 7, y preguntas sociales : todas no;

**Buena** : 8 a 15 y preguntas sociales : todas no;

**Regular**: 16 a 24 y preguntas sociales : 2 si; o Buena más preguntas sociales: 1 sí;

**Malo**: 25 a 32 y preguntas sociales : 2 si; o Regular más preguntas sociales: 3 sí;

**Muy malo**: Malo y preguntas sociales: 3 o más sí.

**APENDICE 6**

**VALORACIÓ GLOBAL DEL PACIENT SOBRE ELS SEUS SIMPTOMES**

**Com completar la escala analògica visual:**

- Si us plau, llegiu les preguntes que se li formularan a continuació amb molta atenció.
- A cada una de les escales que es mostren abaix, inserti una sola ratlla vertical que creui l’escala en el punt que reflexi la situació dels seus símptomes durant la setmana pasada.

1.- ¿Com ha estat la flatulència (ventositats) que ha presentat durant la setmana pasada?

Cap Molt lleu Lleu Moderada Important Molt important

2.- ¿Com ha estat la distensió abdominal que ha presentat durant la setmana pasada?

Cap Molt lleu Lleu Moderada Important Molt important

3.- ¿Com ha estat el malestar/dolor abdominal que ha presentat durant la setmana pasada?

Cap Molt lleu Lleu Moderat Important Molt important

4.- ¿Com han estat les alteracions presentades en la regularitat del seu hàbit intestinal (diarrea) durant la setmana pasada?

Cap Molt lleus Lleus Moderades Importants Molt importants

**APÉNDICE 7**.

**HOJA DE INFORMACIÓN AL PACIENTE**

ENTEROPATIA LINFOCITICA CON SEROLOGIA DE CELIAQUIA NEGATIVA Y REPUESTA CLINICA E HISTOLOGICA A LA DIETA SIN GLUTEN: ESTUDIO CLINICO ALEATORIZADO DE REINTRODUCCION DE GLUTEN vs PLACEBO.

**Código del estudio: EL-DSG/07/2011**

**Estimado paciente:**

Está usted invitado a participar en un estudio clínico cuyo propósito es valorar la posibilidad de reintroducir el gluten en su dieta.

Con este documento pretendemos explicarle los objetivos y procedimientos de este estudio. Por favor, léalo con mucha atención. Si desea realizar alguna pregunta o hay algo que no entienda, consulte al médico responsable del estudio.

###### PROPÓSITO e información general

Usted presenta una forma leve de enteropatía sensible al gluten motivo por el que recibe una dieta sin gluten, y se ha considerado que padece una forma leve de enfermedad celiaca. Usted ya sabe que el gluten es una proteína contenida en algunos cereales (trigo, centeno, cebada y avena). La intolerancia al gluten de la dieta produce una enfermedad que en su forma más habitual produce atrofia de las vellosidades intestinales y se llama enfermedad celíaca. En el adulto es frecuente la presentación como lesiones leves en el intestino en forma de inflamación de la mucosa intestinal (enteritis linfocítica) que es el tipo de sensibilidad al gluten que Usted presenta.

Sin embargo, hay estudios recientes que muestran que existe otra forma de intolerancia al gluten no celiaca, en la cual disminuir la ingesta del gluten mejora los síntomas de la enfermedad, pero en la que probablemente no sería necesario hacer una dieta estricta durante toda la vida. También podría pasar que este tipo de intolerancia al gluten fuera reversible, es decir, que Usted pudiera volver a comer alimentos con gluten sin problemas. Hoy no tenemos pruebas diagnósticas disponibles en la práctica clínica habitual para identificar que tipo de intolerancia al gluten padece Usted.

Con este estudio queremos estudiar si Usted presenta una forma de intolerancia al gluten celiaca o no celiaca, y si persiste esta sensibilidad al gluten que le provocaba síntomas o ha desaparecido. Para ello le proponemos participar en una intervención dietética en que valoraremos que pasa al reintroducir el gluten en comparación con un placebo en 20 pacientes con su enfermedad. Es decir, se le asignará a uno de los 2 grupos de la intervención dietética según un protocolo a doble ciego; esto significa que ni usted ni su médico sabrán cuál de las formulaciones le ha tocado tomar. En este sentido Usted continuará con su dieta sin gluten y recibirá 1 sobre cada 12 horas con gluten o con placebo, en forma de un polvo que deberá mezclar con los alimentos (yogurt, sopas o purés).

La duración del tratamiento será de 6 meses. Durante este tiempo, tendrá que visitar a su médico en 5 ocasiones. Se le pedirá que guarde y rellene cuidadosamente un diario durante la totalidad del estudio; además, deberá llevarlo consigo en cada visita. En este diario tendrá que documentar algunos parámetros específicos de la enfermedad, tales como: número de deposiciones acuosas/blandas/sólidas, dolor y retortijones abdominales, administración regular de los sobres del estudio, ingesta/uso de otros medicamentos.

Tanto al inicio como al final del estudio se le practicará una analítica de sangre y una gastroscopia con biopsias intestinales (con sedación si usted lo considera necesario) para valorar la lesión intestinal. En estas muestras estudiaremos además parámetros inmunológicos que pueden ayudar a diferenciar entre las formas de intolerancia al gluten celiaca y no celiaca.

Debemos informarle de que es posible que su enfermedad empeore tras la reintroducción del gluten aunque esto no hará más que demostrar su grado de sensibilidad al gluten. Si esto se produce antes de los 6 meses, finalizaremos el estudio en este momento (como mínimo Usted deberá de participar en la intervención dietética durante 1 mes). En cambio si su enfermedad no empeora completará el período de 6 meses estipulado.

###### BENEFICIOS POTENCIALES

Este estudio puede ayudar a determinar si Usted presenta una intolerancia al gluten celiaca o no celiaca y, en este sentido, si esta intolerancia persiste y si la dieta sin gluten debe continuar siendo estricta y de por vida.

Durante el estudio, se le realizarán controles periódicos; además, la supervisión médica será más estricta que cuando se utilizan procedimientos terapéuticos normales.

Su participación en este estudio ayudará a que los médicos puedan investigar parámetros analíticos de tipo inmunológico en la biopsia intestinal que en un futuro podrían permitir diferenciar entre estas formas celiacas y no celiacas de intolerancia al gluten.

###### COSTOS DERIVADOS DE LA PARTICIPACIÓN EN EL ESTUDIO

El promotor del estudio (servicio de digestivo) abonará todos los procedimientos médicos relacionados con el mismo, incluidos los sobres de la intervención dietética, de modo que, para Usted, la participación es gratuita.

###### CONFIDENCIALIDAD

Este estudio cumple con las normativas de protección de datos vigentes en España. Todos los datos obtenidos durante el mismo concernientes a su persona serán registrados y científicamente evaluados sin revelar su identidad. A fin de supervisar y comprobar que el estudio se está realizando de conformidad con las normativas legales, es necesario que representantes de las autoridades sanitarias locales o nacionales o representantes de los Comités Éticos examinen sus registros médicos y toda la información obtenida durante el estudio. Estas personas deben respetar las normas de confidencialidad. Sus datos personales son estrictamente confidenciales y no se divulgarán públicamente.

###### NATURALEZA VOLUNTARIA DE LA PARTICIPACIÓN Y EL ABANDONO DEL ESTUDIO

Es importante que comprenda que su participación en este estudio es voluntaria. Si no desea tomar parte, no está obligado a hacerlo. Puede abandonar el estudio en cualquier momento sin que ello implique desventaja alguna en relación con su tratamiento médico habitual o su relación con el médico o el hospital. Si desea abandonar el estudio, póngase en contacto con su médico antes de hacerlo.

Debe solicitar toda la información que desee antes de firmar el formulario de consentimiento informado.

Su médico puede suspender la intervención dietética en cualquier momento si considera que es lo mejor para usted o si ello es necesario para la adecuada realización del estudio (p.ej., si el paciente no cumple con los requisitos del estudio). Si usted lo consiente, se informará a su médico de cabecera sobre su participación en este estudio. La decisión de poner fin a la totalidad del estudio prematuramente es prerrogativa del promotor y del experto médico del promotor, así como de la autoridad competente.

No puede participar en este estudio si ha participado en otro estudio durante los 30 días previos a la visita inicial, si tiene planeado participar en otro estudio simultáneamente o si ya ha participado en este estudio.

**¡Gracias por su colaboración!**

**Si desea obtener más información sobre este estudio o aclarar algún asunto legal, o en caso de emergencia, póngase en contacto con:**

|  |
| --- |

Sello del médico (investigador)

**APÉNDICE 8.**

CONSENTIMIENTO INFORMADO

ENTEROPATIA LINFOCITICA CON SEROLOGIA DE CELIAQUIA NEGATIVA Y REPUESTA CLINICA E HISTOLOGICA A LA DIETA SIN GLUTEN: ESTUDIO CLINICO ALEATORIZADO DE REINTRODUCCION DE GLUTEN vs PLACEBO.

Yo, .......................................................................................................

(nombre y apellidos en Mayúsculas)

- - - He leído la hoja de consentimiento que se me ha entregado.
    - He podido hacer preguntas sobre el estudio
    - He recibido suficiente información sobre el estudio
    - He hablado con:

(nombre del investigador en Mayúsculas)

........................................................................................................

Comprendo que:

1. Mi participación es voluntaria
2. Que este análisis no me supone ningún beneficio directo
3. Que la no aceptación de participar en este estudio no repercutirá en mis cuidados médicos.
4. Que la información obtenida en este estudio es confidencial

(Fecha) (Firma del participante)
